# Heteroligand α-Diimine-Zn(II) Complexes with O,N,O′- and O,N,S-Donor Redox-Active Schiff Bases: Synthesis, Structure and Electrochemical Properties

**DOI:** 10.3390/molecules27238216

**Published:** 2022-11-25

**Authors:** Ivan V. Smolyaninov, Andrey I. Poddel’sky, Daria A. Burmistrova, Yulia K. Voronina, Nadezhda P. Pomortseva, Vasiliy A. Fokin, Ekaterina D. Tselukovskaya, Ivan V. Ananyev, Nadezhda T. Berberova, Igor L. Eremenko

**Affiliations:** 1 Department of Chemistry, Astrakhan State Technical University, 16 Tatisheva Str., 414056 Astrakhan, Russia; 2 G.A. Razuvaev Institute of Organometallic Chemistry, Russian Academy of Sciences, 49 Tropinina Str., 603137 Nizhny Novgorod, Russia; 3 Kurnakov Institute of General and Inorganic Chemistry, Russian Academy of Sciences, Leninskii prospekt 31, 119071 Moscow, Russia; 4 Faculty of Chemistry, National Research University Higher School of Economics, 20 Myasnitskaya Str., 101000 Moscow, Russia

**Keywords:** redox-active ligand, tridentate Schiff base, zinc, crystal structure, X-ray, cyclic voltammetry

## Abstract

A number of novel heteroligand Zn(II) complexes (**1**–**8**) of the general type (L^n^)Zn(NN) containing O,N,O′-, O,N,S-donor redox-active Schiff bases and neutral N,N′-chelating ligands (NN) were synthesized. The target Schiff bases L^n^H_2_ were obtained as a result of the condensation of 3,5-di-*tert*-butyl-2-hydroxybenzaldehyde with substituted *o*-aminophenols or *o*-aminothiophenol. These ligands with combination with 2,2′-bipyridine, 1,10-phenanthroline, and neocuproine are able to form stable complexes upon coordination with zinc(II) ion. The molecular structures of complexes **4**∙H_2_O, **6**, and **8** in crystal state were determined by means of single-crystal X-ray analysis. In the prepared complexes, the redox-active Schiff bases are in the form of doubly deprotonated dianions and act as chelating tridentate ligands. Complexes **6** and **8** possess a strongly distorted pentacoordinate geometry while **4**∙H_2_O is hexacoordinate and contains water molecule coordinated to the central zinc atom. The electrochemical properties of zinc(II) complexes were studied by the cyclic voltammetry. For the studied complexes, O,N,O′- or O,N,S-donor Schiff base ligands are predominantly involved in electrochemical transformations in the anodic region, while the N,N′-coordinated neutral nitrogen donor ligands demonstrate the electrochemical activity in the cathode potential range. A feature of complexes **5** and **8** with sterically hindered *tert*-butyl groups is the possibility of the formation of relatively stable monocation and monoanion forms under electrochemical conditions. The values of the energy gap between the boundary redox orbitals were determined by electrochemical and spectral methods. The parameters obtained in the first case vary from 1.97 to 2.42 eV, while the optical bang gap reaches 2.87 eV.

## 1. Introduction

The formation of organic/organoelemental hybrid molecules containing several types of redox-active ligands requires the development of new approaches characterized by a high efficiency and suitability for preparation of substances with desired properties. One of the promising directions in the synthesis of coordination compounds is the assembly of target complexes via the combination of various redox-active molecular blocks. To solve this problem, it is important to combine several different types of ligands, for example, O,N,O′-, O,N,S-tridentate Schiff bases and N,N′-chelating ligands.

Zinc compounds are the basis for creating substances with light-emitting properties [1]. Zinc complexes represent one of the unique classes of fluorescent compounds that have wide biological applications [2]. Zinc complexes with O,N,O′-donor ligands possess antibacterial, antioxidant, and antitumor activity [3,4,5,6]. At the same time, mixed-ligand zinc complexes with bipyridine ligands and diketonates, tropolones are also characterized by the high cytotoxicity against cancer cells [7,8]. Such interest in the combination of bioactive metal-zinc and natural compounds (curcumin, hydroxyflavones, etc.), as well as molecules with an extended planar aromatic system, which are suitable candidates for intercalation into the DNA structure, is caused by the high antitumor activity of such substances [9]. In particular, zinc complexes with phenanthroline and Schiff bases exhibit cytotoxicity and selectivity towards cancer cells, and are also characterized by the possibility of forming reactive oxygen species and inducing lipid peroxidation [10]. In addition to cytotoxic properties, zinc(II) compounds with various ligands exhibit antioxidant and luminescent properties [11,12].

Along with neutral nitrogen-containing ligands, bi- (O,N-, S,N-), tridentate (O,N,O′-, O,N,N′-, etc.) ligands are widely used for the design of zinc(II) compounds with luminescent and fluorescent properties [13,14,15,16,17,18]. A sufficient number of complex compounds containing transition metals are known For O,N,S-coordinated Schiff bases [19,20,21,22,23]. For zinc derivatives, mainly bis-chelate complexes with N,S-ligands were obtained [24,25,26]. Only a few examples of zinc complexes with tridentate O,N,S-ligands are known [27,28,29]. The introduction of donor/acceptor substituents and the variation of heteroatoms in Schiff bases will affect the spectral and electrochemical properties. Due to the limited valence states available for the metal under study (zinc), the combination of zinc ions with molecular blocks capable of changing their oxidation state will increase the number of possible redox forms of the complexes which in turn can lead to the appearance of new properties.

**Scheme 1:**
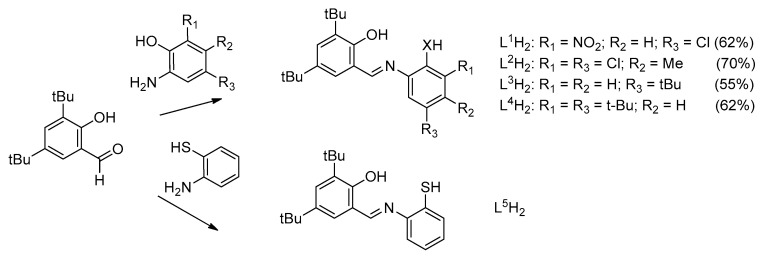
Synthesis of Schiff bases L^1−5^H_2_.

In this regard, the aim of the work is the synthesis of new heteroligand zinc complexes containing neutral N,N′-chelating ligands, such as 2,2′-bipyridine (Bipy), 9,10-phenanthroline (Phen), 2,9-Dimethyl-1,10-phenanthroline (Neocuproine, Neo) and redox-active tridentate O,N,O′- or O,N,S-donor Schiff bases L^n^H_2_ (Scheme 1).

The presence of diverse redox-active ligands determines the possibility of their participation in electrochemical transformations, depending on their ability to donate/accept electrons. A distinctive feature of the synthesized zinc(II) complexes is the absence of the necessity to involve metal in a chemical process since redox-active ligands can serve as a reservoir for storing or transferring electrons. Moreover, there are ample opportunities for fine-tuning the redox properties of ligands by varying substituents. The Schiff bases used in the present work were obtained by the condensation of 3,5-di-*tert*-butyl-2-hydroxybenzaldehyde with functionalized *o*-aminophenols and *o*-aminothiophenol. They are able to form stable complex compounds upon coordination with zinc ions. The presence of substituents stabilizing various redox forms in the structure of ligands makes it possible to evaluate the stability of the oxidized/reduced forms of their complexes using electrochemical methods and to determine the energy gap between the boundary redox orbitals.

## 2. Results and Discussion

### 2.1. Synthesis and Characterization

The reaction of 3,5-di-*tert*-butyl-2-hydroxybenzaldehyde with *o*-aminophenols with various donor or acceptor substituents as well as with *o*-aminothiophenol in refluxing methanol leads to the formation of Schiff bases L^1^H_2_-L^5^H_2_ (Scheme 1).

The preparative yield of L^1^H_2_-L^4^H_2_ was from 55% to 70%. Compounds have been characterized by means of ^1^H and ^13^C{^1^H} NMR-, IR-spectroscopy, and mass-spectrometry.

**Scheme 2:**
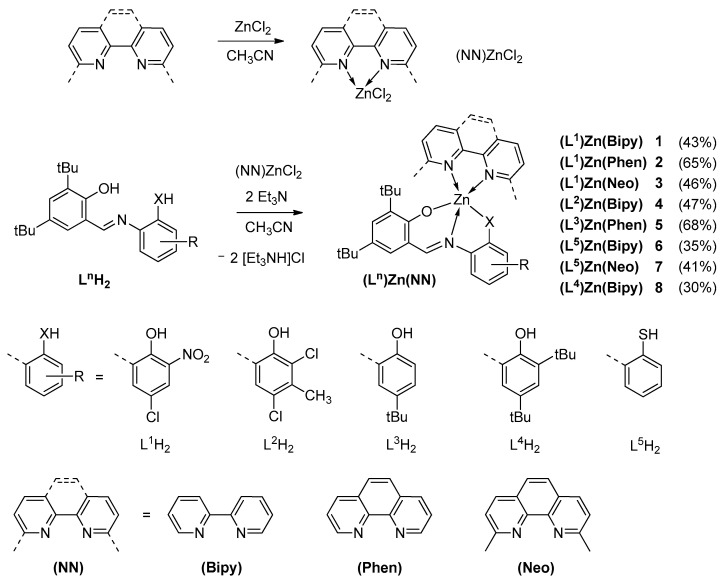
Synthesis of zinc complexes **1**–**8** of the type (L^1−5^)Zn(NN).

Synthesis of zinc(II) complexes **1**–**8** was carried out as the exchange reaction between the corresponding Schiff base and ZnCl_2_ in the presence of α-diimines and triethylamine as deprotonated agent (Scheme 2).

The synthesis was performed in anaerobic conditions to prevent side oxidation processes of ligands. Compounds **1**–**8** were isolated as orange or red-orange crystalline powders with yields up to 68%. Complexes **1**–**8** are air-stable in the solid state. Compounds have been characterized by means of IR, ^1^H and ^13^C{^1^H} NMR spectroscopy (Figures S1–S15), C,H,N-elemental analysis. The spectral and elemental analysis data confirm the composition of complexes. The X-ray suitable crystals of **6**, **8**∙CH_3_CN were grown by the prolonged crystallization of the corresponding powders from acetonitrile solutions under reduced pressure, while the crystals **4**∙H_2_O∙CH_3_CN—by the slow evaporation of acetonitrile on air.

### 2.2. X-ray Structures

**Figure 1:**
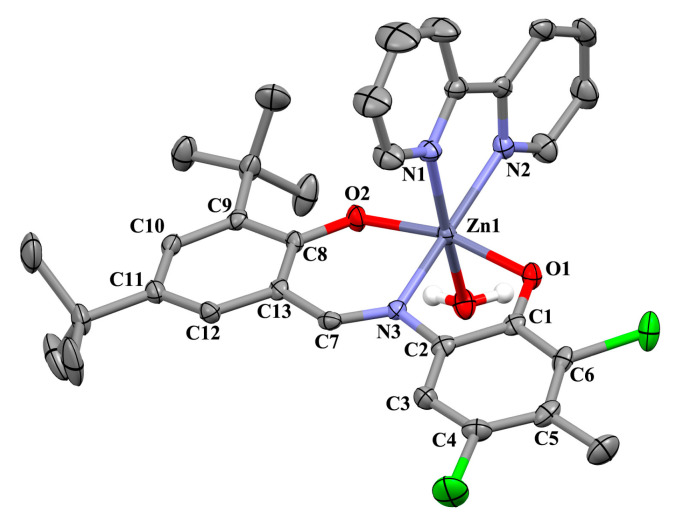
The molecular structure of **4**∙H_2_O in crystals **4**∙H_2_O∙CH_3_CN. The hydrogen atoms except for water are omitted for clarity. The ellipsoids of 50% probability.

**Figure 2:**
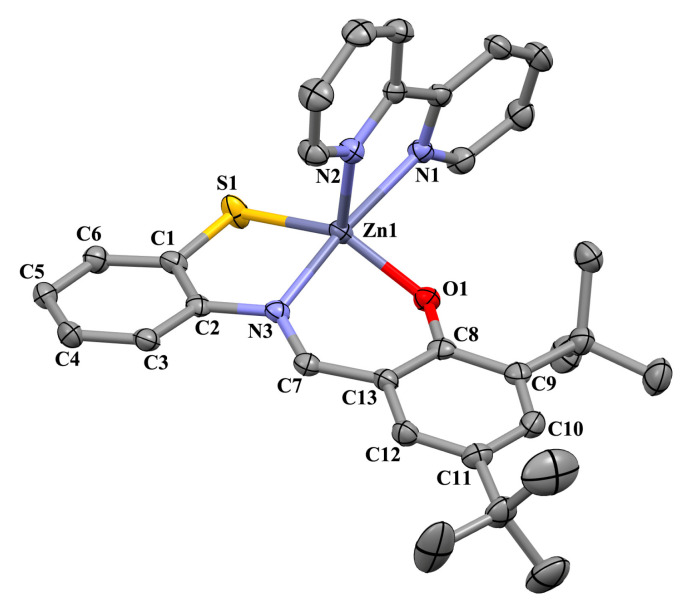
The X-ray structure of **6**. The hydrogen atoms are omitted for clarity. The ellipsoids of 50% probability.

**Figure 3:**
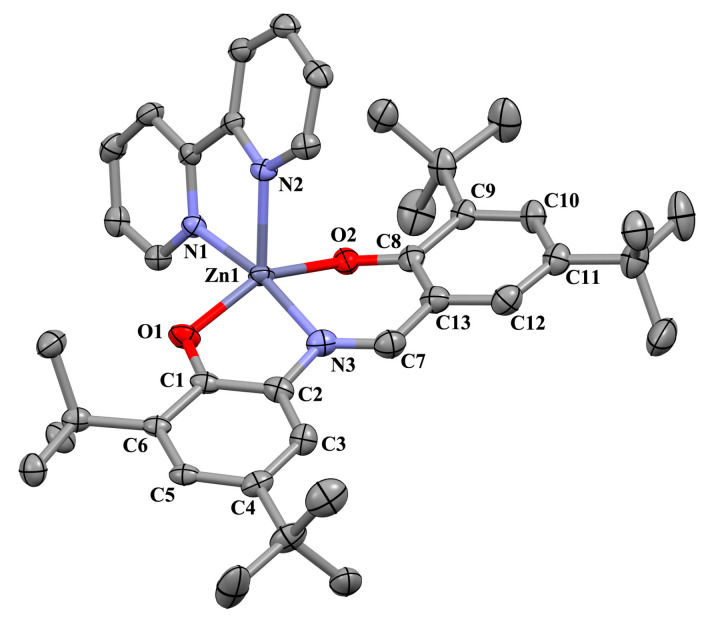
The X-ray structure of **8**. The hydrogen atoms are omitted for clarity. The ellipsoids of 50% probability.

The molecular structures of complexes **4**∙H_2_O (Figure 1), **6** (Figure 2), and **8** (Figure 3) in crystals (**4**∙H_2_O∙CH_3_CN, **6**, and **8**∙CH_3_CN) were determined by using single crystal X-ray diffraction method. The crystal cell of **6** contains two crystallographically independent molecules **6**A and **6**B with similar characteristics, and below we consider **6**A only.

The O,N,O′- or O,N,S-tridentate ligands in complexes contain two six-membered rings, which are linked by the C=N double bond. The ring C(1–6) in complexes **4**∙H_2_O, **6**, and **8** is aromatic with the average C–C bond length of 1.397, 1.395 and 1.402 Å, respectively (Table 1). The second ring C(8-13) is more distorted with av. C–C bond of 1.409, 1.408 and 1.405 Å, respectively. The C7–N3 bonds of 1.298(4), 1.292(4) and 1.279(4) Å are double in conjugation with C(8–13) carbon ring. The C–O bonds—C1–O1 (1.311(3) Å) and C2–O8 (1.294(3) Å) in **4**∙H_2_O; C8–O1 (1.310(4) Å) in **6**; C1–O1 (1.326(3) Å) and C2–O8 (1.307(3) Å) in **8**—are ordinary and correspond to the related C–O bonds in different chelated phenolato complexes [30,31,32,33,34,35]. In general, these bond lengths correspond to these bonds in different zinc complexes with related O,N,O′- or O,N,S-tridentate ligands: the C–O bonds in such complexes are in the range of 1.300–1.325 Å in five-membered metallocycle and 1.315–1.350 Å in six-membered metallocycles [36,37,38,39,40,41,42]. The C–N distances in the earlier reported complexes are 1.280–1.300 Å. The Zn–O and Zn–N bonds in **4**∙H_2_O, **6**, and **8** also typical for such zinc compounds (Zn–O, 1.960–2.105 Å; Zn–N, 2.020–2.090 Å). However, we should to note that the overwhelming number of zinc complexes with these O,N,O′- or O,N,S-tridentate ligands are bi- and polynuclear, where the oxygen of phenolate group behaves as a µ-oxo linker. As a result, the Zn–O and C–O bonds of such moiety are elongated, being closer to the upper limits of the indicated ranges of these bond distances.

Crystals of complex **4** were grown from acetonitrile solution on air as **4**∙H_2_O∙CH_3_CN, where a water molecule is coordinated to the central zinc atom (Figure 1).

The Zn1 atom in **4** adopts a distorted octahedral environment. The central Zn1 atom is disposed slightly of the planes of the chelating ligand: the distance “Zn1…plane O1C(1-6)N3” is 0.052 Å, and the distance “Zn1…plane O2C(8–13)C1N3” is 0.179 Å. The chelating cycles are not planar: the bent angle of the 5-membered chelate cycle along line O1…N3 is 3.26°, while the bent angle of the 6-membered chelate cycle nearly along line O2…N3 is 8.38°.

Complexes **6** and **8** possess a strongly distorted pentacoordinate geometry of the coordination center, which is intermediate between trigonal bipyramid and tetragonal pyramid based on the formal parameter *τ* (*τ* = 1 for ideal trigonal bipyramid and *τ* = 0 for an ideal tetragonal pyramid [43]): *τ* = 0.52 and 0.56 for **6**A and **6**B, and *τ* = 0.29 for **8**. The atoms O1, S1 in **6** or the atoms O1, O2 in **8**, and one of the nitrogen atoms N2 of the N,N′-bipyridine lie in the equatorial plane of the complex molecule. 

**Figure 4:**
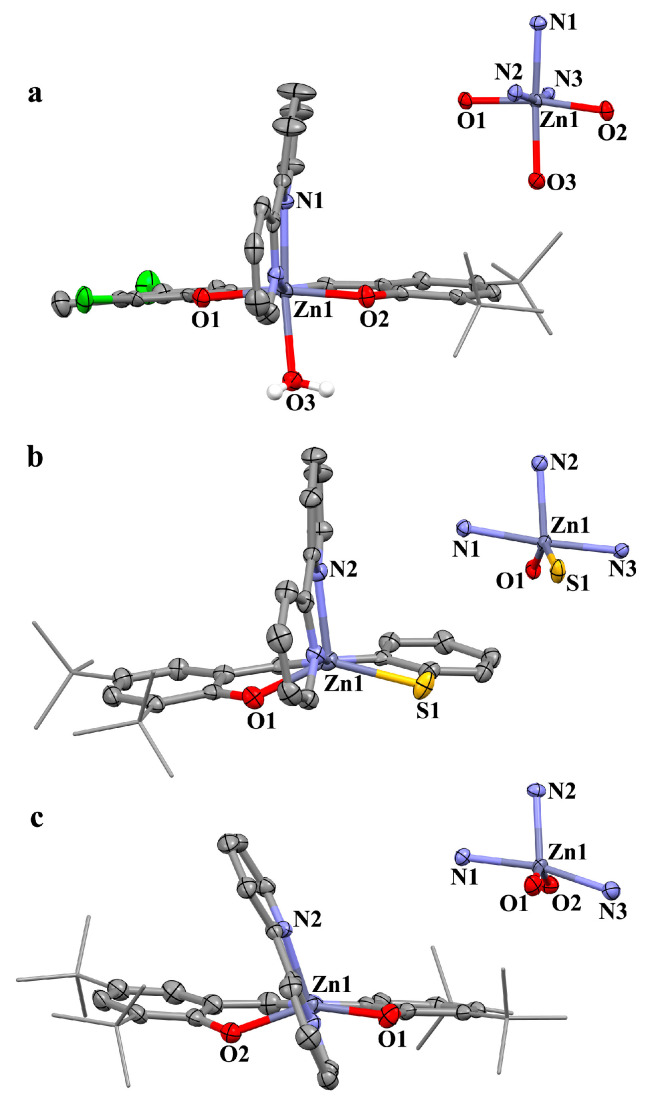
The views on the molecules and inner coordination spheres of **4**∙H_2_O (**a**), **6** (**b**), and **8** (**c**) along the bond Zn1–N3. The ellipsoids of 50% probability. The hydrogen atoms are omitted (except for H_2_O in **4**∙H_2_O), and the tert-butyl groups are given as capped sticks.

The tridentate ligands in these pentacoordinate complexes **6** and **8** are distorted more significantly as compared with hexacoordinated complex **4**∙H_2_O (Figure 4). The central zinc atoms are located out of the planes of chelating fragments of tridentate ligands: the distance “Zn1…plane S1C(1–6)N3” is 0.436 Å, the distance “Zn1… plane O1C(8–13)C7N3” is 0.613 Å in **6**A, and, respectively, 0.143 Å, 0.664 Å in **8**. The angle between the planes formed by the atoms O1, C8, C13, C7, N3 and by the atoms O1, N3, Zn1 (which may be considered as the bent angle of the 6-membered chelate cycle along line O1…N3) is 22.43° in **6**A, that is close to the corresponding angle 24.00° between the planes formed by the atoms O2, C8, C13, C7, N3 and by the atoms O2, N3, Zn1 in **8**. At the same time, the angle between the planes formed by the atoms S1, C1, C2, N3 and by the atoms S1, N3, Zn1 (which may be considered as the bent angle of the 5-membered chelate cycle along line S1…N3) in **6**A is 15.78°, which is remarkably more than the related angle between the planes formed by the atoms O1, C1, C2, N3 and by the atoms O1, N3, Zn1 in **8** of 6.52°.

The presence of a large number of the centres of hydrogen binding leads to the fact that the main structural units in crystal **4** are centrosymmetric H-dimers (Table S1) connected by π…π interactions into infinite chains that form a three-dimensional grid due to weak CH…O and CH… π interactions (Figure S16). There are no classical hydrogen binding donors in the compound **6**, therefore, the main structure–forming interaction in these crystals are π…π, leading to the formation of ribbons interconnected by weak van der Waals interactions (Figure S17). Compound **8** not only lacks hydrogen binding donors, but also the formation of π…π interactions is hindered by volumetric tert-butyl substituents. The crystals of this compound are formed only due to weak CH…O and CH…π interactions and consist of corrugated layers parallel to the c0b plane (Figure S18).

### 2.3. Cyclic Voltammetry

The electrochemical behaviour of zinc complexes **1**–**8** was studied by cyclic voltammetry in dichloromethane solutions using a GC working electrode (Table 2). 

#### 2.3.1. Oxidation

**Figure 5:**
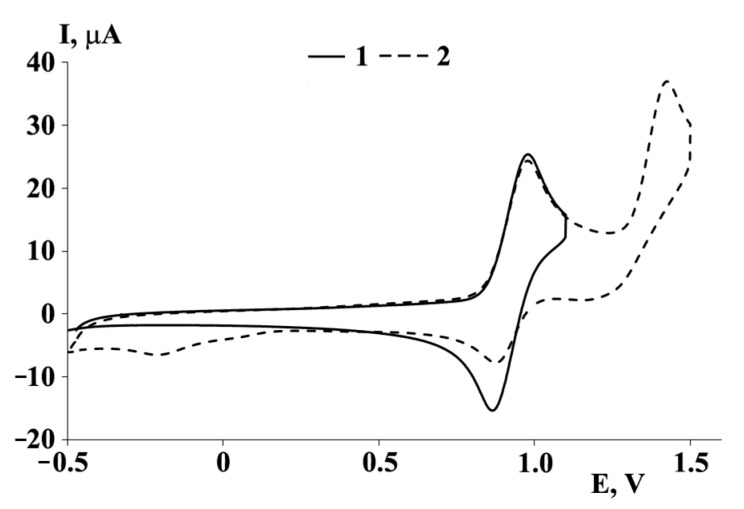
Cyclic voltammograms of **3**: the potential switch from −0.50 to 1.10 V (curve 1); the potential switch from −0.5 to 1.5 V (curve 2) (CH_2_Cl_2_, C = 2 mmol, 0.1 M TBAP, scan rate 200 mV∙s^−1^).

**Figure 6:**
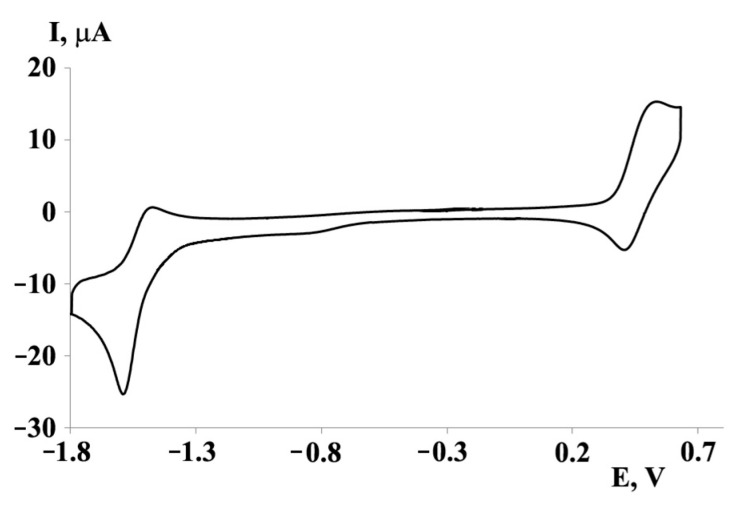
Cyclic voltammogram of **5** the potential switch from 0.63 to -1.80 V (CH_2_Cl_2_, C = 2 mmol, 0.1 M TBAP, scan rate 200 mV∙s^−1^).

The electrochemical oxidation of complexes **1**–**8** proceeds in two or three successive anodic stages (Figure 5). For complexes **1**–**3**, **5**, and **8**, the first anodic peak is quasi-reversible and one-electron, indicating the formation of monocationic particles relatively stable over the CV experiment time (Figure 6, Figures S19, S21 and S27).

**Scheme 3:**
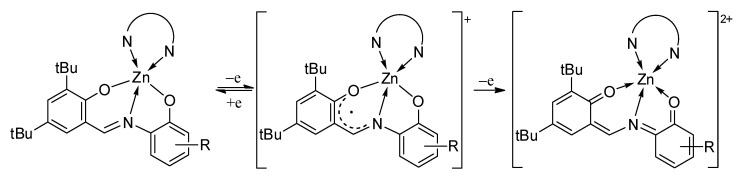
Two successive electrochemical oxidation stages for complexes **1**–**3**, **5**, and **8**.

Based on the values of current ratios, monocationic complexes formed during the electrooxidation of compounds **3** and **8**, containing substituents in the positions of 3,5-aromatic rings in Schiff bases, have the highest stability. The second oxidation stage for complexes **1**–**3**, **5**, and **8** is irreversible, which suggests the formation of an unstable dication intermediate. An increase in the potential sweep range with the capture of the second anodic peak leads to a decrease or complete loss of the reversibility of the first oxidation stage (Figure 5 and Figure S21), which indicates the occurrence of a chemical stage—partial decoordination of the doubly oxidized form of the ligand (Scheme 3).

**Figure 7:**
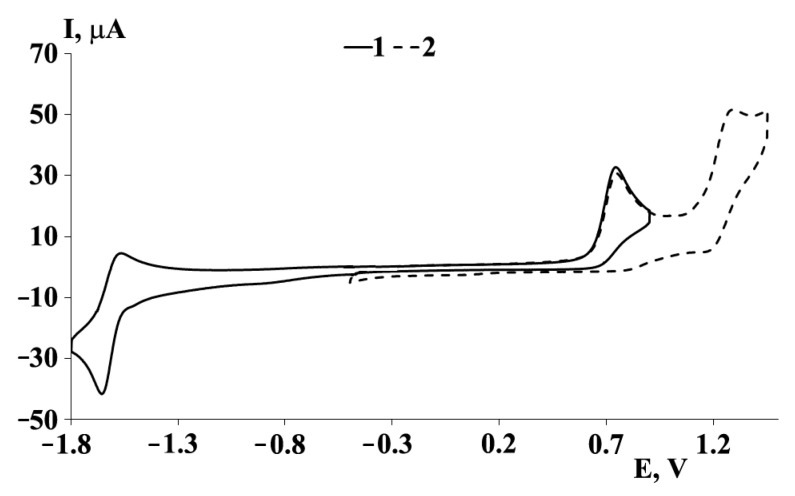
Cyclic voltammograms of **7**: the potential switch from −1.80 to 0.90 V (curve 1); the potential switch from −0.50 to 1.45 V (curve 2) (CH_2_Cl_2_, C = 2 mmol, 0.1 M TBAP, scan rate 200 mV∙s^−1^).

Replacing the O,N,O′-ligand with O,N,S-ligand (in compounds **6**, **7**) or the nitro group with a chlorine atom (in complex **4**) contributes to the destabilization of the monocationic complex generated during electrooxidation, which is expressed in the irreversibility of the first stage of oxidation (Figure 7, Figures S22 and S25). The second anodic peak for compounds **4** and **6** is also irreversible. The presence of a pronounced reversibility for the second anodic peak (I_c_/I_a_ = 0.6) on the CV of complex **7** distinguishes it from other compounds.

**Scheme 4:**
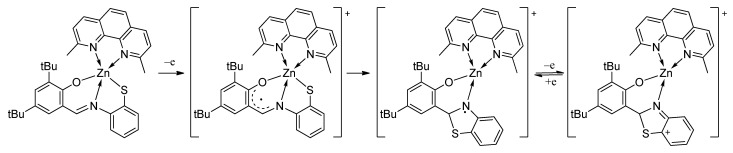
Two electrochemical oxidation of **7** with the intramolecular cyclization stage.

This behaviour can be explained by the occurrence of an intramolecular cyclization reaction in the coordination sphere of the metal in **7** with the formation of a heterocyclic radical (Scheme 4).

Stabilization of this type of intermediate can be achieved due to the presence of a neocuproine ligand in **7**, since complex **6** with the Bipy ligand does not exhibit such behaviour. For Schiff bases acting as ligands, electrically induced cyclization is a typical reaction [44,45]. The ratio of currents for the second stage is less than 1, which implies the occurrence of the subsequent chemical stage, i.e., deprotonation of the tertiary carbon atom with the formation of benzothiazole. Complexes **4** and **5** are characterized by additional anodic steps in the potential range from 1.55 to 1.62 V, which are comparable with the second oxidation potentials (1.56–1.66 V) of free ligands, which may indicate the decoordination of Schiff bases and subsequent reactions deeper oxidation.

#### 2.3.2. Reduction

**Figure 8:**
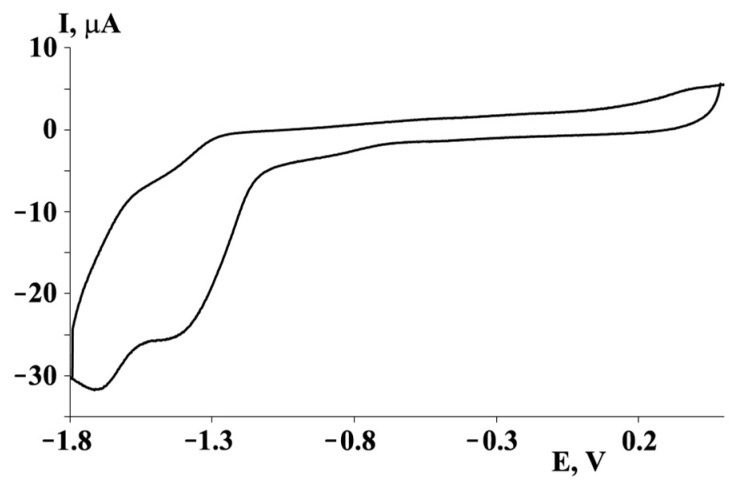
Cyclic voltammogram of **3** the potential switch from 0.50 to −1.80 V (CH_2_Cl_2_, C = 2 mmol, 0.1 M TBAP, scan rate 200 mV∙s^−1^).

For complexes **1**–**3**, several peaks are recorded in the cathodic region in the range from −1.30 to −1.74 V (Figure 8, Figures S20 and S21). In view of the presence of an electroactive NO_2_ group, the reduction mechanism of these compounds becomes much more complicated.

**Figure 9:**
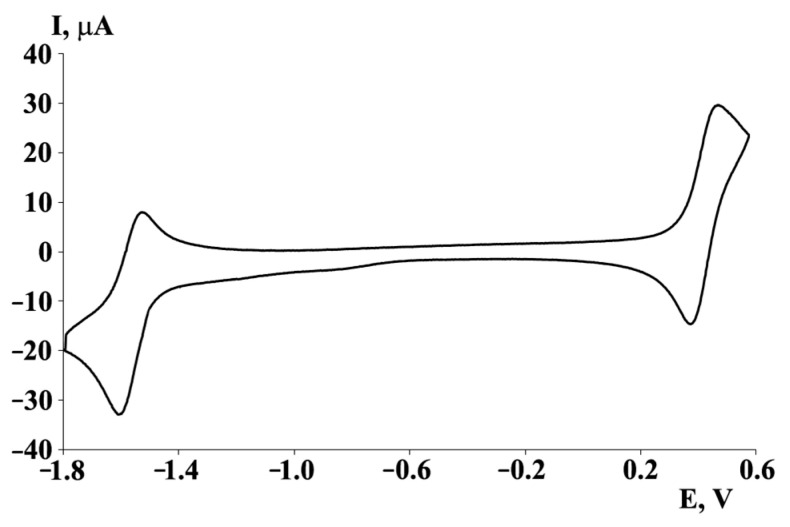
Cyclic voltammogram of **8** the potential switch from 0.55 to −1.80 V (CH_2_Cl_2_, C = 2 mmol, 0.1 M TBAP, scan rate 200 mV∙s^−1^).

For compounds **1** and **2**, a weakly resolvable pre-peak at −1.30 V is observed, which presumably corresponds to the reduction of the nitro group. For a free ligand, a similar redox process is also irreversible, but it is observed at a potential of −0.70 V. Taking into account the presence of the dianionic form of the ligand in **1** and **2** and the expected shift of reduction potentials to the cathodic region, it can be assumed that the nitro group is involved in this electrode process. The additional cathode peaks observed in the range of −1.40 to −1.50 V are in good agreement with the previously obtained results for zinc complexes with coordinated bipyridyl or phenanthroline and an additional S-donor ligand [46,47]. The coordination of the azomethine group at the zinc atom also allows us to consider the possibility of its participation in the cathode stage, however, then one would expect fairly close values of the reduction potentials in a series of complexes **1**–**3**. However, a rather large scatter of potential values is observed. For most other complexes, the first reduction step is quasi-reversible and one-electron (e.g., complexes **8**, **4** and **6**, Figure 9, Figures S23 and S26, respectively).

**Scheme 5:**
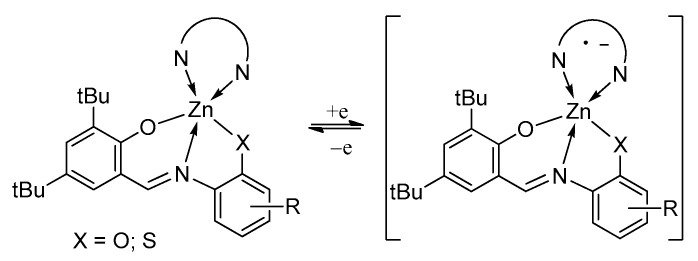
The electrochemical formation of monoanionic derivatives.

As a result of the electrochemical reduction stage, a relatively stable monoanionic complex is formed (Scheme 5). Based on the ratios of the currents, the most stable monoanions are generated during the electroreduction of complexes **6**–**8**.

In the case of complexes **1** and **4**, the proximity of the peak potentials (−1.49 and −1.51 V) indicates the participation of bipyridyl in the cathode stage, while for compounds **6** and **8**, the E^red1^_1/2_ values are shifted to the cathodic region (Table 2). The observed regularities suggest the presence of mutual influence of two types of ligands. The sensitivity of the values of the reduction potentials depending on the O,N,O’- or O,N,S-donor ligand is fixed, for example, for complexes **4** and **6**, where the E^red1^_1/2_ value for complex **6** is shifted to the cathode region up to −1.56 V.

The nature of the substituents in the redox-active Schiff base influences the values of the oxidation potentials. In the case of **1** and **4**, the replacement of the electron-withdrawing nitro group by a chlorine atom with the simultaneous introduction of a methyl substituent leads to a decrease in the oxidation potential by 0.17 V, as well as the presence of two donor tert-butyl groups in **8**. A more pronounced effect is observed for pairs **2** and **5**, or **1** and **8**, for which there is a synchronous shift of E^ox1^_1/2_ and E^ox2^ to the cathode region. For complexes **1** and **2**, the transition from bipyridyl to phenanthroline ligand does not affect the value of the oxidation potential. At the same time, for complex **3**, this value shifts to the anode region, as well as the reduction potential. This fact indicates the presence of a weak electronic interaction between two redox-active ligands. A distinctive feature of compounds **5** and **8** containing donor tert-butyl groups in the O,N,O′-ligand is the stabilization of the monocationic and monoanionic forms of the complexes. The Schiff base takes part in the electrooxidation, while the neutral nitrogen-containing ligand is involved to a greater extent in the cathodic process. An analysis of the potential values suggests that, on the one hand, these complexes act as electron donors and are easily oxidized, and on the other hand, they are weak electron acceptors. This dual behaviour allows us to consider them as electronic reservoirs, which makes it possible to use this type of complexes to initiate photoinduced redox transformations or as electrocatalysts.

One of the main indicators used to assess the possibility of using substances in photovoltaic devices is the energy gap (ΔE), which is the difference in the energies of the boundary orbitals. The value of ΔE can be determined theoretically using quantum chemical calculations or measured experimentally using electrochemistry or UV-visible spectroscopy. Electrochemical methods are widely used to determine the electrochemical gap ΔE_el_ since the values of the standard reduction and oxidation potentials correlate with the LUMO and HOMO energies. In the case of most of the studied complexes, this value was determined as the difference in the potentials of the peaks (Table 2). The values of ΔE_el_ range from 1.97 to 2.42 eV, while the minimum values were obtained for complexes **5** and **8**. The sequential replacement of electron-withdrawing groups in the Schiff base with donor ones leads to a decrease in the value of ΔE_el_ in a row of **1**, **4**, **8**, or for **2** and **5**. This effect is observed mainly due to a decrease in the value of the first oxidation potential due to the involvement of the coordinated Schiff base in the redox processes. In the series of complexes **1**–**3**, the ΔE_el_ parameter is not significantly affected by the auxiliary neutral ligand.

### 2.4. UV-Vis Spectroscopy and DFT Calculations

**Figure 10:**
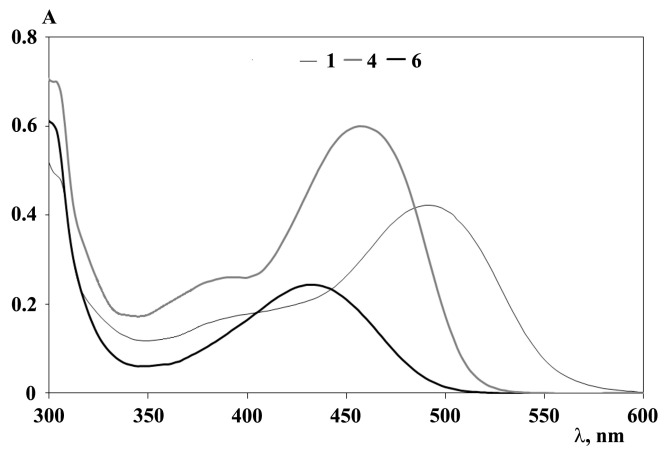
The UV-vis spectra of complexes **1**, **4**, **6** in CHCl_3_ at 293 K (C = 2.5 × 10^−5^ mol/L).

According to the literature data, an overestimation of ΔE_el_ compared to the spectral value (ΔE) is typical for polymers and crystalline materials, while the opposite effect is recorded for low molecular weight compounds [48]. As a result, we have studied the activity of the complexes in the UV-visible range of the spectrum (280–600 nm) in chloroform (Table 3). For the investigated zinc(II) complexes in this spectral range, two or three absorption bands are observed with an intensity maximum in the region of 430–500 nm (Table 3, Figure 10).

The largest shift of the absorption maximum to the long wavelength region is characteristic of complexes **1**–**3** with an electron-withdrawing nitro group. The introduction of donor substituents into the Schiff base, on the contrary, promotes a hypsochromic shift of λ_max_ in the visible region of the spectrum for complexes **4**, **5**, and **8**. The replacement of an oxygen atom by sulfur in the case of compounds **6** and **7** has a similar effect on the position of the absorption maximum. The calculated values of the energy gap for the complexes are observed in a narrow range from 2.49 to 2.87 eV. The closer values of ΔE, which are consistent with the results of measuring ΔE_el_, were obtained only for the first three zinc(II) complexes. In other cases, the ΔE values have higher values than those obtained on the basis of the electrochemical studies.

**Figure 11:**
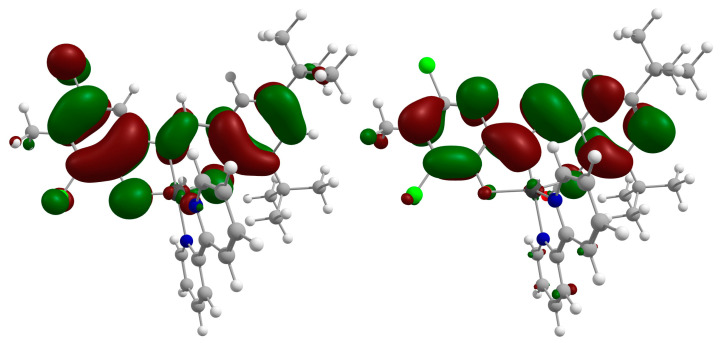
The isosurfaces of HOMO (**left**) and LUMO+1 (**right**) in the optimized structure of **4**.

**Figure 12:**
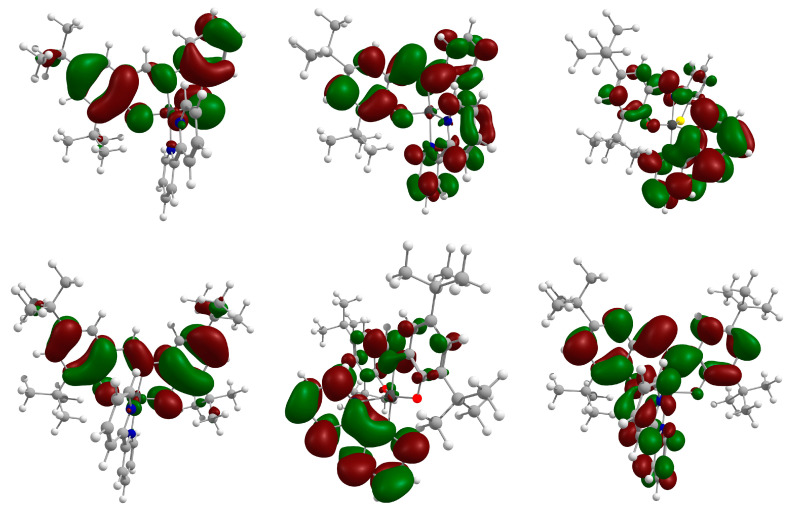
The isosurfaces of HOMO (**left**), LUMO+1 (**middle**) and LUMO+2 (**right**) orbitals for the optimized structures of **6** (**top**) and **8** (**bottom**).

The density functional theory calculations were performed for molecules **4**, **6** and **8** at the PBE0-D3/6-311++G(d,p) level in CHCl_3_ solution (the SCRF-PCM model) to unambiguously describe the observed spectral data. Noteworthy, while the geometry optimization led to insignificant changes in the structures of **6** and **8** molecules as compared to those in crystals (rmsd 0.1022 and 0.087 Å, respectively), the resulting structure of **4**·H_2_O (Figure S28) is characterized by the pentacoordinate metal atom with the water molecule participating only in a moderate hydrogen bond with the oxygen O2 atom (O3…O2 2.722 Å, the O2-H-O3 angle equals to 165.0°). The solution of the time-dependent Kohn-Sham equations for the optimized structures with the accounting of non-equilibrium PCM solvation reasonably describes corresponding spectral data in the long-wave regions. Namely, the excitation energies for the simplest allowed transitions having the largest oscillator strength values do correspond to the λ_Max_ values with a rather good accuracy (452, 425, and 460 nm for **4**, **6,** and **8**, respectively). A closer look at these transitions revealed that neither of them involves the metal atom region. In **4**, the transition corresponds to the intraligand excitation from the π-bonding (HOMO) to the π* orbital (LUMO+1) both centered on the Schiff ligand (Figure 11). Note that the HOMO orbital also covers regions of the lone electronic pairs on chlorine atoms while the LUMO+1 orbital does not; hence, the corresponding transition can be described as the combination of n-π and π-π intraligand charge transfers. For **6** and **8**, the interligand charge transfer is observed that corresponds to the excitations from the same HOMO π-bonding orbital of the Schiff base to the LUMO+1 and LUMO+2 π-antibonding orbitals centered on both ligands (Figure 12). The additional calculations for the other complexes under study demonstrated a similar order of molecular orbitals (Figures S29 and S30): for instance, the HOMO is always a π-bonding orbital located on the Schiff base, and the LUMO is located on a neutral NN ligand. This allows to assume that the discussed excitation mechanisms could be a common feature for this class of compounds.

## 3. Materials and Methods

### 3.1. General

The starting reagents were commercially available ZnCl_2_ (anhydrous, «Ekros», 99%, Saint Petersburg, Russia), 3,5-di-*tert*-butylsalicylic aldehyde («Acros Organics», 99%, Shanghai, China), 2-amino-4-*tert*-butylphenol («Alfa Aesar», 97%, Heysham, England), 2-amino-4-chloro-6-nitrophenol («Aldrich», 97%, Burlington, MA, USA), 6-amino-2,4-dichloro-3-methylphenol hydrochloride (Fluka, 97%, Steinheim, Germany), 2-aminothiophenol (≥97%, Hohenbrunn, Germany), 2,2′-bipyridine (98%, Sigma Aldrich, Saint Louis, MO, USA), 1,10-phenanthroline (99%, Alfa Aesar, Shanghai, China), neocuproine hemihydrate (99+%, Acros Organics, Waltham, MA, USA) tetrabutylammonium perchlorate (Bu_4_NClO_4_) («Alfa Aesar», 99%, China) were used without further purification. 4,6-Di-*tert*-butyl-*o*-aminophenol was synthesized by the known method [49]. Solvents were purified following standard procedures [50].

The IR spectra were recorded on an FSM-1201 FT-IR spectrometer (LLC “Monitoring”, Saint Petersburg, Russia) in KBr pellets. The NMR spectra were measured in CDCl_3_ on Bruker Avance HD 400 spectrometers (Bruker Biospin AG, Faellanden, Switzerland) with a frequency of 400 MHz (1H) and 100 MHz (^13^C) using Me_4_Si as an internal standard. The chemical shift values are given in ppm with reference to the solvent, and the coupling constants (J) are given in Hz. The elemental analysis was carried out on a Euro EA 3000 (C,H,N) elemental analyzer (EuroVector Srl, Redavalle, Italy). Mass spectra (HRMS) were recorded on a Bruker UHR-TOF Maxis™ Impact mass spectrometer (Bruker Daltonik GmbH, Bremen, Germany). The UV-VIS spectra were recorded with a SF-104 spectrophotometer (AKVILON, Podol’sk, Russia) in a range of 300–600 nm. 

### 3.2. Synthesis and Characterization

#### 3.2.1. General Method for Preparation of Tridentate Schiff Bases

Reactions of 3,5-di-tert-butyl-2-hydroxybenzaldehyde (4 mmol) with o-aminophenols or o-aminothiophenols (4 mmol) were carried out in methanol (20 mL). The appropriate aminothiophenol (in 1 mL methanol) was added dropwise to a solution of aldehyde (in 20 mL) for 30 min, and then the solution was refluxed for 5 h under argon. The solution was slowly cooled to room temperature. The resulting precipitate was filtered off and dried under reduced pressure. The Schiff bases such as 2-((E)-(2-Mercaptophenylimino)methyl)-4,6-di-tert-butylphenol (L^5^H_2_), 2,4-di-tert-butyl-6-(((5-tert-butyl)-2-hydroxyphenyl)imino)methyl)phenol (L^3^H_2_) were synthesized by the known methods [21,51].

#### 3.2.2. L^1^H_2_

Yield 1.14 g, 2.8 mmol (62%). IR (KBr, ν/cm^−1^): 3225, 3082, 2961, 2910, 2870, 1620, 1597, 1580, 1522, 1470, 1435, 1408, 1363, 1310, 1248, 1202. ^1^H NMR (400 MHz, CDCl_3_, δ, ppm): 1.33 (s, 9H, tBu), 1.48 (s, 9H, tBu), 7.24 (d, ^4^J(H,H) = 2.4 Hz, 1H, arom. C_6_H_2_), 7.47 (d, ^4^J(H,H) = 2.5 Hz, 1H, arom. C_6_H_2_), 7.52 (d, ^4^J(H,H) = 2.4 Hz, 1H, arom. C_6_H_2_), 8.01 (d, ^4^J(H,H) = 2.5 Hz, 1H, arom. C_6_H_2_), 8.74 (s, 1H, CH=N), 10.87 (s, 1H, OH), 13.24 (br.s, 1H, OH). ^13^C {^1^H} NMR (100 MHz, CDCl_3_, δ, ppm): 29.38, 31.39, 34.20, 35.16, 117.90, 121.24, 124.88, 127.18, 127.26, 129.51, 134.25, 137.48, 140.83, 140.99, 148.11, 158.86, 166.65. Calcd. for C_21_H_25_ClN_2_O_4_ (%): C, 62.30; H, 6.22; N, 6.92. Found (%): C, 62.18; H, 6.47; N, 7.01. 

#### 3.2.3. L^2^H_2_

Yield 1.14 g, 2.8 mmol (70%). IR (KBr, ν/cm^−1^): 3515, 3532, 3008, 2961, 2910, 2869, 1613, 1580, 1470, 1434, 1395, 1373, 1361, 1334, 1295, 1273, 1251, 1229, 1202. ^1^H NMR (400 MHz, CDCl_3_, δ, ppm): 1.33 (s, 9H, tBu), 1.47 (s, 9H, tBu), 2.48 (s, 3H, CH_3_), 5.97 (br.s, 1H, OH), 7.17 (s, 1H, arom. C_6_H_1_), 7.24 (d, ^4^J(H,H) = 2.4 Hz, 1H, arom. C_6_H_2_), 7.49 (d, ^4^J(H,H) = 2.4 Hz, 1H, arom. C_6_H_2_), 8.70 (s, 1H, CH=N), 12.93 (br.s, 1H, OH). ^13^C {^1^H} NMR (100 MHz, CDCl_3_, δ, ppm): 17.69, 29.39, 31.41, 34.21, 35.12, 117.86, 118.18, 121.96, 125.95, 127.26, 129.03, 132.95, 134.62, 137.24, 141.08, 144.61, 158.21, 165.50. EI-MS: Found *m*/*z*: 408.1498 [M + H]^+^. C_22_H_28_Cl_2_NO_2_. Calcd *m*/*z*: 408.1492.

#### 3.2.4. L^4^H_2_

Yield 1.10 g, 2.5 mmol (62%). IR (KBr, ν/cm^−1^): 3519, 3458, 2957, 2907, 2866, 1615, 1584, 1481, 1468, 1440, 1413, 1392, 1361, 1331, 1271, 1250, 1219,1200. ^1^H NMR (400 MHz, CDCl_3_, δ, ppm): 1.35 (s, 9 H, tBu), 1.36 (s, 9 H, tBu), 1.46 (s, 9 H, tBu), 1.48 (s, 9 H, tBu), 6.23 (br.s, 1 H, OH), 7.02 (d, ^4^J(H,H) = 2.1 Hz, 1 H, arom. C_6_H_2_), 7.28 (d, ^4^J(H,H) = 2.1 Hz, 1 H, arom. C_6_H_2_), 7.30 (d, ^4^J(H,H) = 2.3 Hz, 1 H, arom. C_6_H_2_), 7.50 (d, ^4^J(H,H) = 2.3 Hz, 1 H, arom. C_6_H_2_), 8.70 (s, 1 H, CH=N), 12.69 (br.s, 1 H, OH). ^13^C {^1^H} NMR (100 MHz, CDCl_3_, δ, ppm): 29.40, 29.50, 31.45, 31.62, 34.25, 34.59, 35.05, 35.71, 112.66, 118.63, 123.01, 127.27, 128.70, 135.39, 135.77, 137.25, 141.30, 142.47, 146.12, 157.55, 164.63. EI-MS: Found *m*/*z*: 438.3358 [M + H]^+^. C_29_H_44_NO_2_. Calcd *m*/*z*: 438.3367.

#### 3.2.5. General Method for Preparation of Zinc Complexes **1**–**8**

Synthesis of zinc complexes was carried out as follows: 1 equiv. neutral nitrogen-containing ligand (NN) (0.35 mmol) dissolved in acetonitrile (2 mL) was added to a solution of ZnCl_2_ (0.35 mmol) in the same solvent (5 mL) under argon. As a result of mixing, a suspension of the (NN)ZnCl_2_ complex is formed, then 0.35 mmol of Schiff base and 2 equiv. triethylamine (0.7 mmol) were added under extensive stirring. During the reaction, the initial precipitate of (NN)ZnCl_2_ gradually dissolved and the color of the solution changed to intense orange or brick red. The resulting solution was left for 2 days at 0–4 °C. The resulting colored precipitates of the complexes were filtered off, washed with n-hexane, and dried under reduced pressure. Crystals suitable for X-ray diffraction analysis were obtained by recrystallization of the compounds in acetonitrile.

#### 3.2.6. (L^1^)Zn(Bipy) (1) O,N,O′-(2,4-Di-tert-butyl-6-((5-chloro-3-nitro-2-oxidophenylimino)methyl)phenolato)-N,N′-(2,2′-bipyridyl)zinc(II)

The yield of **1** in the form of red crystals was 43% (0.094 g). IR (KBr, cm^−1^): 3076, 2954, 2903, 2869, 1600, 1543, 1522, 1507, 1473, 1458, 1442, 1385, 1340, 1252, 1215, 1200, 1160, 1131, 1103. ^1^H NMR (400 MHz, CDCl_3_, δ/J, ppm/Hz): 0.98 (s, 9H, tBu), 1.28 (s, 9H, tBu), 7.00 (s, 1H, arom. C_6_H_2_), 7.26 (s, 1H, arom. C_6_H_2_), 7.46 (br.t, J = 5.9 Hz, 2 H, 2,2′-bipy), 7.55 (s, 1H, arom. C_6_H_2_), 7.72 (s, 1H, arom. C_6_H_2_), 7.98 (br.t, J = 6.8 Hz, 2 H, 2,2′-bipy), 8.18 (br.d, J = 5.9 Hz, 2 H, 2,2′-bipy), 8.58 (br.s, 2 H, 2,2′-bipy), 8.67 (s, 1H, CH=N). ^13^C NMR (100 MHz, CDCl_3_, δ, ppm): 28.88, 31.27, 33.83, 35.03, 115.97, 117.89, 118.26, 121.43, 121.45, 123.08, 126.59, 128.59, 129.62, 134.87, 137.93, 140.25, 140.30, 141.27, 141.51, 149.06, 161.68. Calcd. for C_31_H_31_ClN_4_O_4_Zn (%): C, 59.63; H, 5.00; N, 8.97. Found (%): C, 59.84; H, 5.25; N, 8.92. 

#### 3.2.7. (L^1^)Zn(Phen) (2) O,N,O′-(2,4-Di-tert-butyl-6-((5-chloro-3-nitro-2-oxidophenylimino)methyl)phenolato)-N,N′-(1,10-phenanthroline)zinc(II)

The yield of **2** in the form of red powder was 65% (0.147 g). IR (KBr, cm^−1^): 3079, 3059, 2954, 2903, 2873, 1597,1544, 1519, 1458, 1429, 1407, 1386, 1337, 1253, 1198, 1156, 1104. ^1^H NMR (400 MHz, CDCl_3_, δ/J, ppm/Hz): 0.81 (s, 9H, tBu), 1.28 (s, 9H, tBu), 6.96 (d, J = 2.5 Hz, 1H, arom. C_6_H_2_), 7.20 (d, J = 2.5 Hz, 1H, arom. C_6_H_2_), 7.57 (d, J = 2.6 Hz, 1 H, arom. C_6_H_2_), 7.62-7.68 (m, 3H: 1H, arom. C_6_H_2_ + 2H, Phen), 7.94 (s, 2H, Phen), 8.43 (d, J = 8.0 Hz, 2 H, Phen), 8.66 (s, 1 H, CH=N), 8.86 (d, J = 3.9 Hz, 2 H, Phen). ^13^C NMR (100 MHz, CDCl_3_, δ, ppm): 28.72, 31.24, 33.80, 34.89, 116.31, 117.80, 118.27, 122.89, 124.79, 126.83, 128.46, 128.75, 129.51, 134.53, 138.09, 138.68, 141.12, 142.07, 149.10, 157.75, 161.39, 170.91. EI-MS: Found *m*/*z*: 647.1353 [M + H]^+^. C_33_H_32_ClN_4_O_4_Zn. Calcd. *m*/*z*: 647.1398.

#### 3.2.8. (L^1^)Zn(Neo) O,N,O′-(2,4-Di-tert-butyl-6-((5-chloro-3-nitro-2-oxidophenylimino)methyl)phenolato)-N,N′-(2,9-dimethyl-1,10-phenanthroline)zinc(II)

The yield of **3** in the form of red crystals was 46% (0.109 g). IR (KBr, cm^−1^): 3070, 2955, 2903, 2867, 1594, 1572, 1542, 1522, 1507, 1460, 1426, 1410, 1361, 1340, 1255, 1210, 1197, 1160, 1131, 1104. ^1^H NMR (400 MHz, CDCl_3_, δ/J, ppm/Hz): 0.62 (s, 9H, tBu), 1.27 (s, 9H, tBu), 2.76 (s, 6H, CH_3_, Neo), 3.18 (s, 3H, CH_3_, free 1/2Neo), 7.05 (d, J = 2.3 Hz, 1H, arom. C_6_H_2_), 7.20 (d, J = 2.3 Hz, 1H, arom. C_6_H_2_), 7.57 (d, J = 8.3 Hz, 2 H, arom., Neo), 7.62 (d, J = 2.6 Hz, 1H, arom. C_6_H_2_), 7.77 (dd, J = 8.3 Hz, J = 0.8 Hz, 1H, arom., free 1/2Neo), 7.84 (d, J = 0.8 Hz, 2H, arom., Neo), 7.85 (d, J = 2.6 Hz, 1H, arom. C_6_H_2_), 7.91 (d, J = 0.8 Hz, 1H, arom., free 1/2Neo), 8.36 (dd, J = 8.3 Hz, J = 0.8 Hz, 2 H, arom., Neo), 8.47 (dd, J = 8.3 Hz, J = 0.8 Hz, 1H, arom., free 1/2Neo), 8.82 (s, 1 H, CH=N). ^13^C NMR (100 MHz, CDCl_3_, δ, ppm): 25.00, 25.02, 28.39, 31.26, 33.83, 34.63, 115.32, 117.47, 118.01, 122.91, 125.50, 125.75, 125.95, 126.41, 127.00, 127.09, 128.69, 129.57, 134.61, 137.57, 138.98, 139.87, 140.16, 140.74, 140.77, 141.18, 158.73, 158.98, 160.68, 161.30, 171.32. Calcd. for C_42_H_41_ClN_5_O_4_Zn ((L^1^)Zn(Neo)·0.5Neo) (%): C, 64.62; H, 5.29; N, 8.97. Found (%): C, 64.90; H, 5.49; N, 8.78. 

#### 3.2.9. (L^2^)Zn(Bipy) (4) O,N,O′-(2,4-Di-tert-butyl-6-((3,5-dichloro-4-methyl-2-oxidophenylimino)methyl)phenolato)-N,N′-(2,2′-bipyridyl)zinc(II)

The yield of **4** in the form of bright orange crystals was 47% (0.110 g). IR (KBr, cm^−1^): 3076, 2951, 2907, 2870, 1605, 1587, 1520, 1458, 1447, 1407, 1383, 1361, 1316, 1253, 1228, 1196, 1157, 1139. ^1^H NMR (400 MHz, CDCl_3_, δ/J, ppm/Hz): 0.96 (s, 9H, tBu), 1.28 (s, 9H, tBu), 2.38 (s, 3H, CH_3_), 7.01 (d, J = 2.4 Hz, 1H, arom. C_6_H_2_), 7.21 (d, J = 2.4 Hz, 1H, arom. C_6_H_2_), 7.36-7.45 (m, 3 H: 2H, 2,2′-bipy + 1H, arom. C_6_H_1_), 7.90 (td, J = 7.8 Hz, J = 1.0 Hz, 2H, 2,2′-bipy), 8.16 (d, J = 7.9 Hz, 2H, 2,2′-bipy), 8.59 (d, J = 4.6 Hz, 2 H, 2,2′-bipy), 8.69 (s, 1H, CH=N). ^13^C NMR (100 MHz, CDCl_3_, δ, ppm): 18.01, 28.95, 31.39, 33.78, 34.97, 105.73, 112.32, 118.34, 121.43, 125.86, 128.31, 132.18, 134.10, 134.59, 139.75, 139.80, 140.70, 149.03, 149.07, 159.61, 169.73. Calcd. for C_32_H_33_Cl_2_N_3_O_2_Zn (%): C, 61.21; H, 5.30; N, 6.69. Found (%): C, 61.48; H, 5.46; N, 6.51. 

#### 3.2.10. (L^3^)Zn(Phen) (5) O,N,O′-(2,4-Di-tert-butyl-6-((5-tert-butyl-2-oxidophenylimino)methyl)phenolato)-N,N′-(1,10-phenanthroline)zinc(II)

The yield of **5** in the form of orange powder was 68% (0.165 g). IR (KBr, cm^−1^): 3050, 2951, 2902, 2866, 1609, 1593, 1539, 1520, 1486, 1458, 1428, 1382, 1362, 1300, 1273, 1253, 1230, 1196, 1160, 1141, 1127, 1102. ^1^H NMR (400 MHz, CDCl_3_, δ/J, ppm/Hz): 0.81 (s, 9H, tBu), 1.29 (s, 9H, tBu), 1.38 (s, 9H, tBu), 6.78 (d, J = 8.4 Hz, 1H, arom. C_6_H_3_), 7.03 (dd, J = 8.4 Hz, J = 1.7 Hz, 1H, arom. C_6_H_3_), 7.10 (d, J = 2.3 Hz, 1 H, arom. C_6_H_2_), 7.15 (d, J = 2.3 Hz, 1 H, arom. C_6_H_2_), 7.56 (d, J = 1.7 Hz, 1 H, arom. C_6_H_3_), 7.68 (m, 2H, Phen), 7.89 (s, 2H, Phen), 8.38 (d, J = 7.8 Hz, 2 H, Phen), 8.84 (s, 1 H, CH=N), 8.94 (d, J = 3.7 Hz, 2 H, Phen). ^13^C NMR (100 MHz, CDCl_3_, δ, ppm): 28.72, 31.52, 31.88, 33.80, 34.14, 34.84, 108.98, 118.39, 118.92, 124.68, 125.14, 126.66, 127.25, 128.38, 128.65, 133.49, 134.23, 136.25, 138.12, 140.37, 141.33, 149.22, 157.88, 159.44, 168.60. EI-MS: Found *m*/*z*: 624.2575 [M + H]^+^. C_37_H_42_N_3_O_2_Zn. Calcd. *m*/*z*: 624.2563. 

#### 3.2.11. (L^5^)Zn(Bipy) (6) O,N,S-(2,4-Di-tert-butyl-6-((2-sulfidophenylimino)methyl)phenolato)-N,N′-(2,2′-bipyridyl)zinc(II)

The yield of **6** in the form of orange crystals was 35% (0.069 g). IR (KBr, cm^−1^): 3066, 3042, 2954, 2904, 2867, 1603, 1574, 1524, 1490, 1475, 1458, 1439, 1382, 1360, 1330, 1313,1273, 1251, 1236, 1157, 1135, 1101. ^1^H NMR (400 MHz, CDCl_3_, δ/J, ppm/Hz): 1.04 (s, 9H, tBu), 1.28 (s, 9H, tBu), 6.96–7.07 (m, 2H, arom., C_6_H_4_), 7.04 (d, J = 2.4 Hz, 1H, arom. C_6_H_2_), 7.25 (d, J = 2.4 Hz, 1H, arom. C_6_H_2_), 7.41-7.47 (m, 3 H: 2H, 2,2′-bipy + 1H, arom. C_6_H_4_), 7.60 (dd, J = 7.2 Hz, J = 1.8 Hz, 1 H, arom. C_6_H_4_), 7.92 (td, J = 7.8 Hz, J = 1.8 Hz, 2H, arom. 2,2′-bipy), 8.10 (d, J = 8.0 Hz, 2 H, 2,2′-bipy), 8.54 (d, J = 4.4 Hz, 2 H, 2,2′-bipy), 8.63 (s, 1H, CH=N). ^13^C NMR (100 MHz, CDCl_3_, δ, ppm): 28.94, 31.41, 33.76, 35.01, 115.34, 118.51, 121.11, 122.54, 125.91, 126.20, 128.62, 128.85, 133.27, 134.06, 139.53, 140.78, 146.52, 148.77, 149.00, 163.83, 168.65. Calcd. for C_31_H_33_N_3_OSZn (%): C, 66.36; H, 5.93; N, 7.49. Found (%): C, 66.21; H, 6.12; N, 7.39. 

#### 3.2.12. (L^5^)Zn(Neo) (7) O,N,S-(2,4-Di-tert-butyl-6-((2-sulfidophenylimino)methyl)phenolato)-N,N′-(2,9-dimethyl-1,10-phenanthroline)zinc(II)

The yield of **7** in the form of light orange powder was 41% (0.069 g). IR (KBr, cm^−1^): 3048, 2952, 2904, 2866, 1598, 1575, 1522, 1508, 1460, 1426, 1381, 1364, 1332, 1272, 1252, 1235, 1200, 1159, 1138, 1102. ^1^H NMR (400 MHz, CDCl_3_, δ/J, ppm/Hz): 0.69 (s, 9H, tBu), 1.28 (s, 9H, tBu), 2.85 (s, 6H, CH_3_, Neo), 7.00–7.04 (m, 2H, arom., C_6_H_4_), 7.07 (d, J = 2.6 Hz, 1H, arom. C_6_H_2_), 7.21 (d, J = 2.6 Hz, 1H, arom. C_6_H_2_), 7.38–7.44 (m, 1H, arom., C_6_H_4_), 7.54 (d, J = 8.3 Hz, 2 H, arom., Neo), 7.61–7.68 (m, 1H, arom., C_6_H_4_), 7.81 (s, 2H, arom., Neo), 8.28 (d, J = 8.3 Hz, 2 H, arom., Neo), 8.71 (s, 1 H, CH=N). ^13^C NMR (100 MHz, CDCl_3_, δ, ppm): 24.94, 28.55, 31.41, 33.77, 34.57, 116.18, 118.20, 122.37, 125.59, 125.65, 125.90, 127.08, 128.44, 128.87, 132.95, 133.77, 138.14, 140.53, 141.04, 142.75, 146.20, 159.41, 163.65, 169.57. Calcd. for C_35_H_37_N_3_OSZn (%): C, 68.56; H, 6.08; N, 6.85. Found (%): C, 68.63; H, 6.27; N, 6.79. 

#### 3.2.13. (L^4^)Zn(Bipy) (8) O,N,O′-(2,4-Di-tert-butyl-6-((3,5-di-tert-butyl-2-oxidophenylimino)methyl)phenolato)-N,N′-(2,2′-bipyridyl)zinc(II)

The yield of complex in the form of red crystals was 30% (0.069 g). IR (KBr, cm^−1^): 3103, 3069, 2951, 2907, 2870, 1607, 1598, 1578, 1560, 1542, 1522, 1475, 1443, 1409, 1377, 1315, 1297, 1275, 1253, 1230, 1198, 1161. ^1^H NMR (400 MHz, CDCl_3_, δ/J, ppm/Hz): 1.04 (s, 9H, tBu), 1.23 (s, 9H, tBu), 1.31 (s, 9H, tBu), 1.37 (s, 9H, tBu), 7.09 (d, J = 2.4 Hz, 1H, arom. C_6_H_2_), 7.11 (d, J = 2.7 Hz, 1H, arom. C_6_H_2_), 7.19 (d, J = 2.7 Hz, 1 H, arom. C_6_H_2_), 7.19 (d, J = 2.4 Hz, 1 H, arom. C_6_H_2_), 7.48-7.52 (m, 2 H, 2,2′-bipy), 8.01 (t, J = 7.5 Hz, 2H, 2,2′-bipy), 8.13 (d, J = 7.9 Hz, 2H, 2,2′-bipy), 8.65 (d, J = 4.3 Hz, 2 H, 2,2′-bipy), 8.75 (s, 1H, CH=N). Calcd. for C_39_H_49_N_3_O_2_Zn (%):C, 71.27; H, 7.52; N, 6.39. Found (%): C, 71.47; H, 7.68; N, 6.23. 

### 3.3. X-ray Diffraction

The X-ray diffraction data were collected on a D8 Venture automatic diffractometer using graphite monochromated MoKα (λ 0.71073Å) radiation. SADABS program [52] was used to perform absorption correction. All structures were solved by direct methods and refined by full-matrix least-squares using the *SHELXL2018/3* [53] with OLEX2 [54]. All the non-hydrogen atoms were refined with anisotropic atomic displacement parameters. The positions of hydrogen atoms were located from the Fourier electron density synthesis and were included in the refinement in the isotropic riding model approximation.The main crystallographic data and structure refinement details for all complexes are presented in Table S2. CCDC 2209787 (**4**∙H_2_O∙CH_3_CN), 2209788 (**6**), 2212742 (**8**∙CH_3_CN) contain the supplementary crystallographic data. These data can also be obtained free of charge at ccdc.cam.ac.uk/structures/ from the Cambridge Crystallographic Data Centre. 

### 3.4. Cyclic Voltammetry

Electrochemical studies were carried out using VERSASTAT-3 potentiostat (PAR) in three-electrode mode. The stationary glassy carbon (d = 2 mm) disk was used as the working electrode; the auxiliary electrode was the platinum-flag electrode. The reference electrode was Ag/AgCl/KCl (sat.) with a watertight diaphragm. All measurements were carried out under argon. The samples were dissolved in the pre-deaerated solvent. The scan rate (ν) was 200 mV∙s^−1^. The supporting electrolyte 0.1 M Bu_4_NClO_4_ was dried under reduced pressure (48 h) at 50 °C. The concentration of compounds was 1–3 mmol.

### 3.5. Computational Details

The density functional theory calculations were performed using the Gaussian09 program [55]. The PBE0 functional [56,57] and the standard 6-311++G(d,p) basis set was utilized to perform all the calculations. The non-specific solvation effects which can affect molecular properties in a solution were accounted for by means of the SCRF-PCM model [58] (ε = 4.806 for CHCl_3_). The empirical dispersion correction by Grimme [59] with the Becke-Johnson damping scheme [60] were employed for the geometry optimization procedures. The TD-DFT calculations were performed using the first ten singlet states and including SCRF equilibrium correction.

## 4. Conclusions

The interaction of O,N,O-, O,N,S-donor Schiff bases derived from 3,5-di-tert-butyl-2-hydroxybenzaldehyde with ZnCl_2_ and N,N′-diimine ligands leads to the formation of heteroligand zinc(II) complexes with a preparative yield of up to 68%. The molecular structures of compounds **4**·H_2_O, **6**, and **8** in crystal were studied by X-ray diffraction. The data of X-ray diffraction analysis confirm the fact that the Schiff bases in zinc complexes are in the dianionic imino-bis-phenolate or thiophenolate-iminophenolate forms. It is worth noting that the tridentate ligands in the pentacoordinate complexes **6** and **8** are distorted more significantly as compared with the hexacoordinate complex **4**∙H_2_O.

Due to the redox inertness of the zinc(II) ion, the synthesized complexes are convenient objects for studying the electrochemical behavior, since the contribution of different types of ligands to redox transformations can be estimated. Based on the results of CV experiments, complexes **1**–**3** with electron-withdrawing groups in the O,N,O′-donor ligand tend to form relatively stable monocationic complexes containing oxidized forms of Schiff bases. In the cathodic area for these compounds, the reduction proceeds in irreversible manner and involves both the redox-active nitro-group and the neutral N,N′-donor ligand. The replacement of an oxygen atom by sulfur in complexes **6** and **7** or the introduction of chlorine substituents into the aromatic ring of O,N,O′-redox-active ligand in the case of compound **4** contributes to the destabilization of the electrogenerated oxidized forms. The reversibility of the second oxidation stage for complex **7** can be explained by the intramolecular cyclization reaction with the formation of a heterocyclic radical coordinated at the zinc atom.

Compounds **4**–**8** are characterized by the participation of neutral N,N′-diimine ligands in redox transformations with the formation of stable monoanion species upon electroreduction. A general tendency for the heteroligand zinc complexes is the separation of ligand functions: in the anodic region, coordinated Schiff bases are predominantly involved in redox processes, while in the cathodic region, neutral N,N′-diimine ligands are participated. A specific feature of compounds **5** and **8** is the possibility to form the relatively stable monocations and monoanions, which makes it possible to consider these objects as potential reservoirs for electron storage and transfer. Variation in the substituents nature in the Schiff bases affects the oxidation potentials and, accordingly, the reducing activity of this type of complexes. On the one hand, the above complexes are electron donors and are easily oxidized, and on the other hand, they act as weak electron acceptors. This dual behavior causes the use of this type complex to initiate photoinduced redox transformations or as electrocatalysts.

It was found that the variation of the substituents nature in the Schiff base has the greatest effect on the value of ΔE_el_ than the size of the N,N′-diimine aromatic system. The ΔE_el_ values range from 1.97 to 2.42 eV. The minimum values were obtained for complexes **5** and **8** capable of forming stable oxidized/reduced forms. The substitution of electron-withdrawing groups by donor ones in the redox-active ligand promotes a decrease in the first oxidation potential for compounds **4**, **5**, and **8** and, consequently ΔE_el_ is lowered to 1.97 eV. The calculated values of the energy gap ΔE, determined on the basis of spectral data, are characterized by slightly overestimated values (2.49–2.87 eV) compared to the values of ΔE_el_, which is typical of low molecular weight compounds.

## Figures and Tables

**Table 1:** The selected bond lengths (Å) in **4**∙H_2_O, **6**, and **8** according to the X-ray analysis data.

Bond	4∙H_2_O	6A/6B	8
Zn1–N1	2.189(2)	2.181(3)/2.158(3)	2.109(2)
Zn1–N2	2.148(2)	2.128(3)/2.151(3)	2.100(2)
Zn1–N3	2.086(2)	2.084(3)/2.079(3)	2.062(2)
Zn1–O1	2.0634(19)	1.951(2)/1.952(3)	1.985(2)
Zn1–O2	2.0055(19)	-	1.998(2)
Zn1–O3	2.238(2)	-	-
Zn1–S1	-	2.3149(10)/2.3219(10)	-
C1–O1	1.311(3)	-	1.326(3)
C8–O2	1.294(3)	-	1.307(3)
C8–O1	-	1.310(4)/1.299(4)	-
C2–N3	1.412(4)	1.422(4)/1.413(5)	1.426(4)
C7–N3	1.298(4)	1.292(4)/1.292(5)	1.279(4)
C1–S1	-	1.766(4)/1.769(4)	-
C1–C2	1.425(4)	1.404(5)/1.415(5)	1.408(4)
C1–C6	1.411(4)	1.402(5)/1.395(5)	1.427(4)
C2–C3	1.387(4)	1.399(5)/1.389(5)	1.392(4)
C3–C4	1.382(4)	1.387(5)/1.389(5)	1.392(4)
C4–C5	1.390(4)	1.391(5)/1.388(6)	1.399(4)
C5–C6	1.386(4)	1.388(5)/1.383(5)	1.396(4)
C7–C13	1.433(4)	1.434(5)/1.438(5)	1.442(4)
C8–C9	1.443(4)	1.440(5)/1.440(5)	1.439(4)
C8–C13	1.434(4)	1.431(5)/1.431(5)	1.435(4)
C9–C10	1.384(4)	1.376(5)/1.383(5)	1.388(4)
C10–C11	1.412(4)	1.422(5)/1.404(5)	1.408(4)
C11–C12	1.369(4)	1.361(5)/1.369(5)	1.367(4)
C12–C13	1.413(4)	1.417(5)/1.419(5)	1.395(4)

**Table 2:** The CV data for **1**–**8** (CH_2_Cl_2_, GC, 0.1M nBu_4_ClO_4_, C = 2 ×10^−3^ M, Ar, vs. Ag/AgCl/KCl (sat.)).

Compound	E^ox1^_1/2_, V	I_c_/I_a_	E^ox2^, V	E^red1^_1/2_, V	I_a_/I_c_	E^red2^_p_, V	ΔE_el_, eV
**1**	0.87	0.7	1.40	−1.49 *	-	−1.74 *	2.42
**2**	0.87	0.8	1.23	−1.50 *	-	−1.72 *	2.44
**3**	0.92	1.0	1.43	−1.40 *	-	−1.70 *	2.38
**4**	0.70 *	-	0.86	−1.48	0.4	-	2.18
**5**	0.46	0.6	1.09	−1.55	0.4	-	2.01
**6**	0.77 *	-	1.10	−1.56	0.8	-	2.38
**7**	0.73 *	-	1.24 **	−1.62	0.7	-	2.38
**8**	0.41	1.0	0.77	−1.56	0.7	-	1.97

*—the peak potential; **—the value of potential E^ox2^_1/2_.

**Table 3:** The UV-vis spectral data for **1**–**8** in CHCl_3_ solution at 293 K.

No.	Compound	λ_Max_, nm (ε, 10^3^ M^−1^cm^−1^)	ΔE, eV
**1**	(L^1^)Zn(Bipy)	305 (16.6), 385 (6.5), 490 (18.7)	2.53
**2**	(L^1^)Zn(Phen)	378 (4.4), 491 (17.1)	2.52
**3**	(L^1^)Zn(Neo)	385 (4.6), 497 (18.0)	2.49
**4**	(L^2^)Zn(Bipy)	304 (28.5), 376 (10.2), 457 (28.8)	2.71
**5**	(L^3^)Zn(Phen)	310 (6.6), 382 (4.7), 448 (10.8)	2.76
**6**	(L^5^)Zn(Bipy)	303 (20.0), 432 (10.1)	2.87
**7**	(L^5^)Zn(Neo)	316 (6.9), 438 (10.6)	2.82
**8**	(L^4^)Zn(Bipy)	307 (9.8), 381 (9.7), 451 (7.7)	2.74

## Data Availability

The data presented in this study are available in this article.

## Supplementary Materials

The following supporting information can be downloaded at: https://www.mdpi.com/article/10.3390/molecules27238216/s1, Figures S1–S15 with NMR data; Figures S16–S18 with crystal packing of **4**∙H_2_O∙CH_3_CN, **6**, and **8**∙CH_3_CN; Figures S19–S27 with CV curves for complexes; Figures S28–S30 with DFT results (General view of the optimized molecular structure of **4**·H_2_O; The isosurfaces of HOMO and LUIMO orbitals in the selected complexes); Table S1 with H-bonds in crystals of investigated compounds; Table S2 with the main crystallographic data and structure refinement details.

## Sample Availability

Samples of the compounds are available from the authors.
