# Supplementary material for: Heteroligand α-Diimine-Zn(II) Complexes with O,N,O′- and O,N,S-Donor Redox-Active Schiff Bases: Synthesis, Structure and Electrochemical Properties
Source: Molecules. 2022 Nov 25;27(23):8216. doi: 10.3390/molecules27238216 (PMC9736860; doi:10.3390/molecules27238216)
Supplement: Supplementary file 1 [file molecules-27-08216-s001.zip › molecules-2030796-supplementary.pdf]

Heteroligand α-diimine-Zn(II) complexes with O,N,O′- and O,N,S- donor redox-active Schiff bases: synthesis, structure and electrochemical properties

Ivan V. Smolyaninov[1,*], Andrey I. Poddel'sky[2], Daria A. Burmistrova[1,*], Yulia K. Voronina[3], N. P. Pomortseva[1], V.A. Fokin[1], Tselukovskaya E.D.[3,4], Ananyev I.V.[3], Nadezhda T. Berberova[1], Igor L. Eremenko[3]

[1] Department of Chemistry, Astrakhan State Technical University, 16 Tatisheva Str., 414056 Astrakhan, Russia
[2] G.A. Razuvaev Institute of Organometallic Chemistry, Russian Academy of Sciences, 49 Tropinina Str., 603137 Nizhny Novgorod, Russia
[3] Kurnakov Institute of General and Inorganic Chemistry, Russian Academy of Sciences, Leninskii prospekt 31, 119071 Moscow, Russia
[4] National Research University Higher School of Economics, 20 Myasnitskaya Str., 101000 Moscow, Russia
* Correspondence: burmistrova.da@gmail.com (D.A.B.); ivsmolyaninov@gmail.com (I.V.S.)

Supplementary materials

Content:

Figure S1. The ^{1}H NMR spectrum of **1** (400 MHz, $CDCl_3$) 3

Figure S2. The $^{13}C\{^{1}H\}$ NMR spectrum of **1** (100 MHz, $CDCl_3$) 3

Figure S3. The ^{1}H NMR spectrum of **2** (400 MHz, $CDCl_3$) 4

Figure S4. The $^{13}C\{^{1}H\}$ NMR spectrum of **2** (100 MHz, $CDCl_3$) 4

Figure S5. The ^{1}H NMR spectrum of **3** (400 MHz, $CDCl_3$) 5

Figure S6. The $^{13}C\{^{1}H\}$ NMR spectrum of **3** (100 MHz, $CDCl_3$) 5

Figure S7. The ^{1}H NMR spectrum of **4** (400 MHz, $CDCl_3$) 6

Figure S8. The $^{13}C\{^{1}H\}$ NMR spectrum of **4** (100 MHz, $CDCl_3$) 6

Figure S9. The ^{1}H NMR spectrum of **5** (400 MHz, $CDCl_3$) 7

Figure S10. The $^{13}C\{^{1}H\}$ NMR spectrum of **5** (100 MHz, $CDCl_3$) 7

Figure S11. The ^{1}H NMR spectrum of **6** (400 MHz, $CDCl_3$) 8

Figure S12. The $^{13}C\{^{1}H\}$ NMR spectrum of **6** (100 MHz, $CDCl_3$) 8

Figure S13. The ^{1}H NMR spectrum of **7** (400 MHz, $CDCl_3$) 9

Figure S14. The $^{13}C\{^{1}H\}$ NMR spectrum of **7** (100 MHz, $CDCl_3$) 9

Figure S15. The ^{1}H NMR spectrum of **8** (400 MHz, $CDCl_3$). 10

Figure S16. The crystal packing of **4**$\cdot H_2O\cdot CH_3CN$. 10

Figure S17. The crystal packing of **6**. 11

Figure S18. The crystal packing of **8**·CH_3CN. 11

Figure S19. Cyclic voltammograms of **1** (L^1)Zn(Bipy) the potential switch from -0.50 to 1.0 V (curve 1); the potential switch from -0.50 to 1.55 V (curve 2) 12

Figure S20. Cyclic voltammogram of **1** (L^1)Zn(Bipy) the potential switch from 0.0 to -1.9 V 12

Figure S21. Cyclic voltammograms of **2** (L^1)Zn(Phen) the potential switch from 1.1 to -1.9 V (curve 1); the potential switch from -0.5 to 1.3 V (curve 2) 13

Figure S22. Cyclic voltammograms of **4** (L^2)Zn(Bipy) the potential switch from -0.45 to 0.80 V (curve 1); the potential switch from -0.45 to 0.95 V (curve 2) 13

Figure S23. Cyclic voltammogram of **4** (L^2)Zn(Bipy) the potential switch from 0.50 to -1.75 V 14

Figure S24. Cyclic voltammograms of **5** (L^3)Zn(Phen) the potential switch from -0.45 to 0.63 V (curve 1); the potential switch from -0.45 to 0.95 V (curve 2) 14

Figure S25. Cyclic voltammograms of **6** (L^5)Zn(Bipy) the potential switch from -0.50 to 0.85 V (curve 1); the potential switch from -0.50 to 1.20 V (curve 2) 15

Figure S26. Cyclic voltammogram of **6** (L^5)Zn(Bipy) the potential switch from 0.50 to -1.80 V 15

Figure S27. Cyclic voltammogram of **8** (L^4)Zn(Bipy) the potential switch from -0.50 to 0.85 V 16

Figure S28. General view of the optimized molecular structure of **4**·H_2O. 16

Figure S29. The isosurfaces of HOMO orbitals in the selected complexes. 17

Figure S30. The isosurfaces of LUMO orbitals in the selected complexes. 17

Table S1. H-bonds in crystals of investigated compounds. 18

Table S2. The details of X-ray experiment and structure refinement. 19

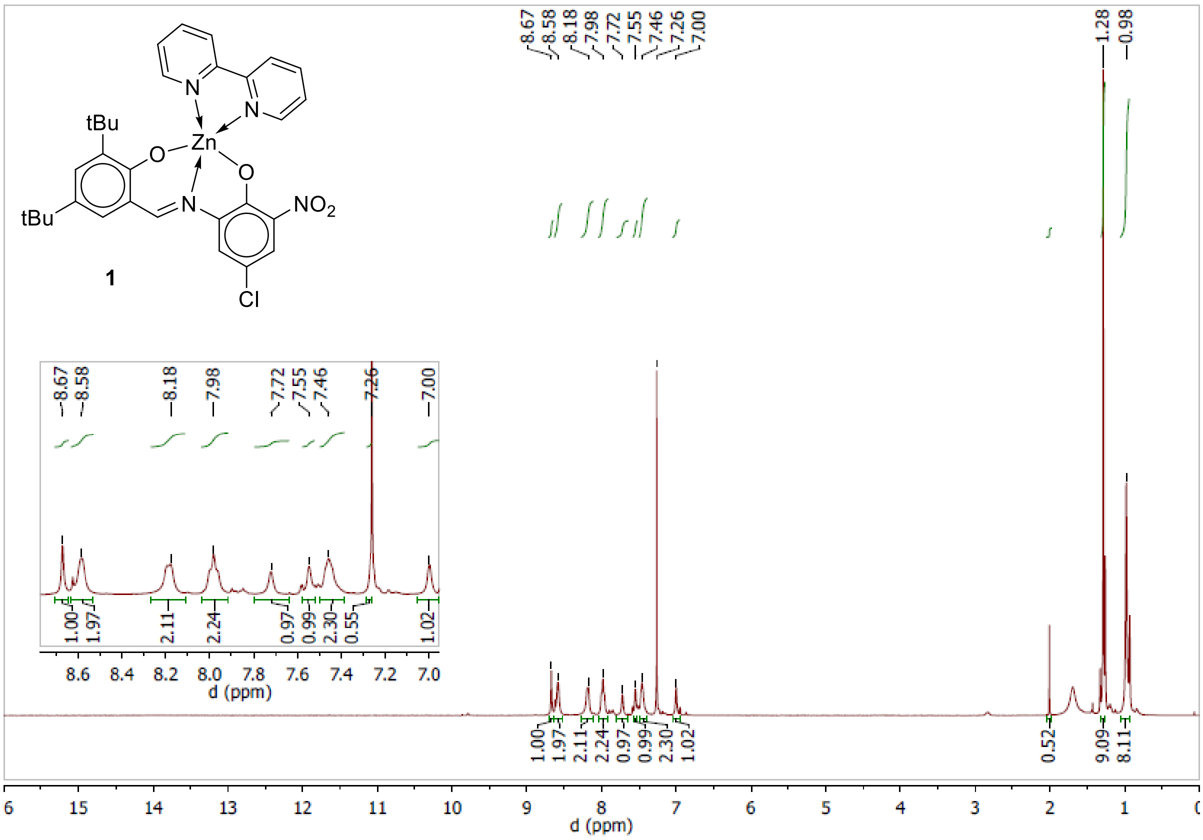

Figure S1. The ^{1}H NMR spectrum of **1** (400 MHz, $CDCl_3$).

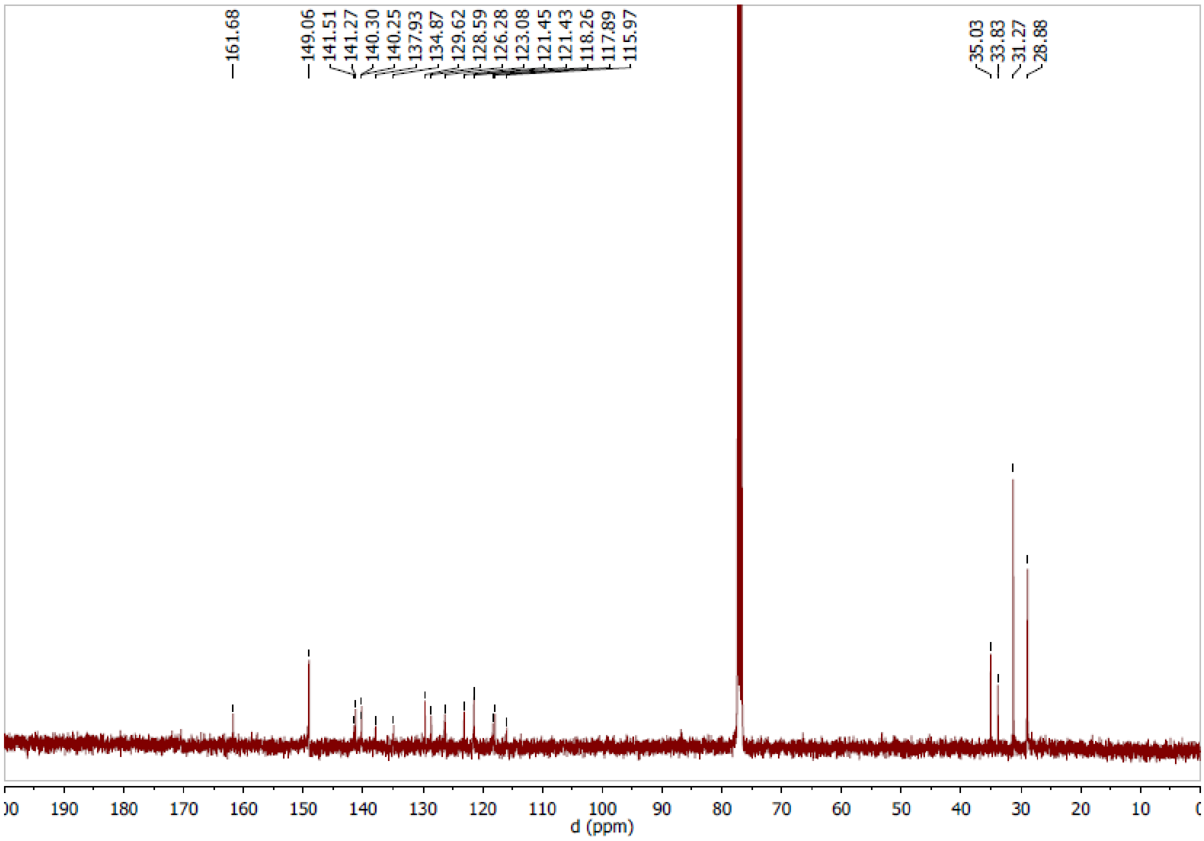

Figure S2. The $^{13}C\{^1H\}$ NMR spectrum of **1** (100 MHz, $CDCl_3$).

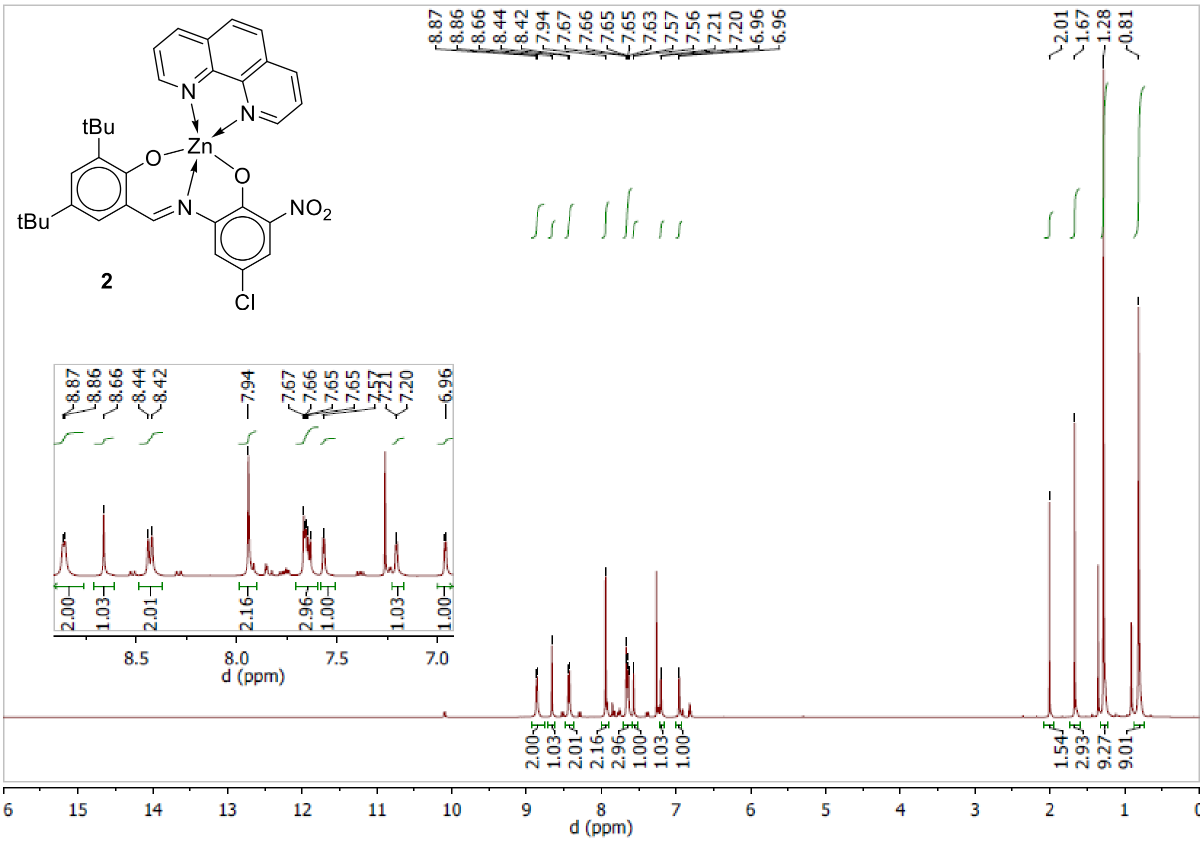

Figure S3. The ^{1}H NMR spectrum of **2** (400 MHz, $CDCl_3$).

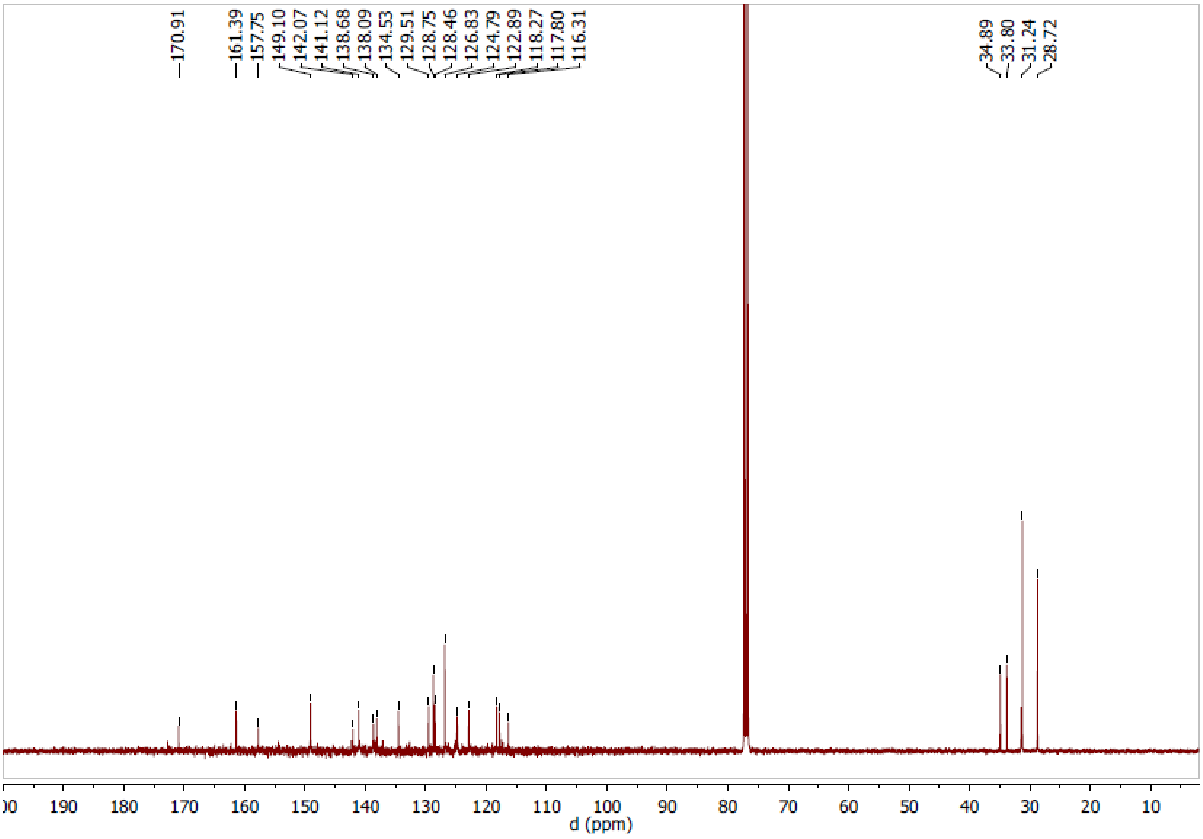

Figure S4. The ^{13}C{^{1}H} NMR spectrum of **2** (100 MHz, $CDCl_3$).

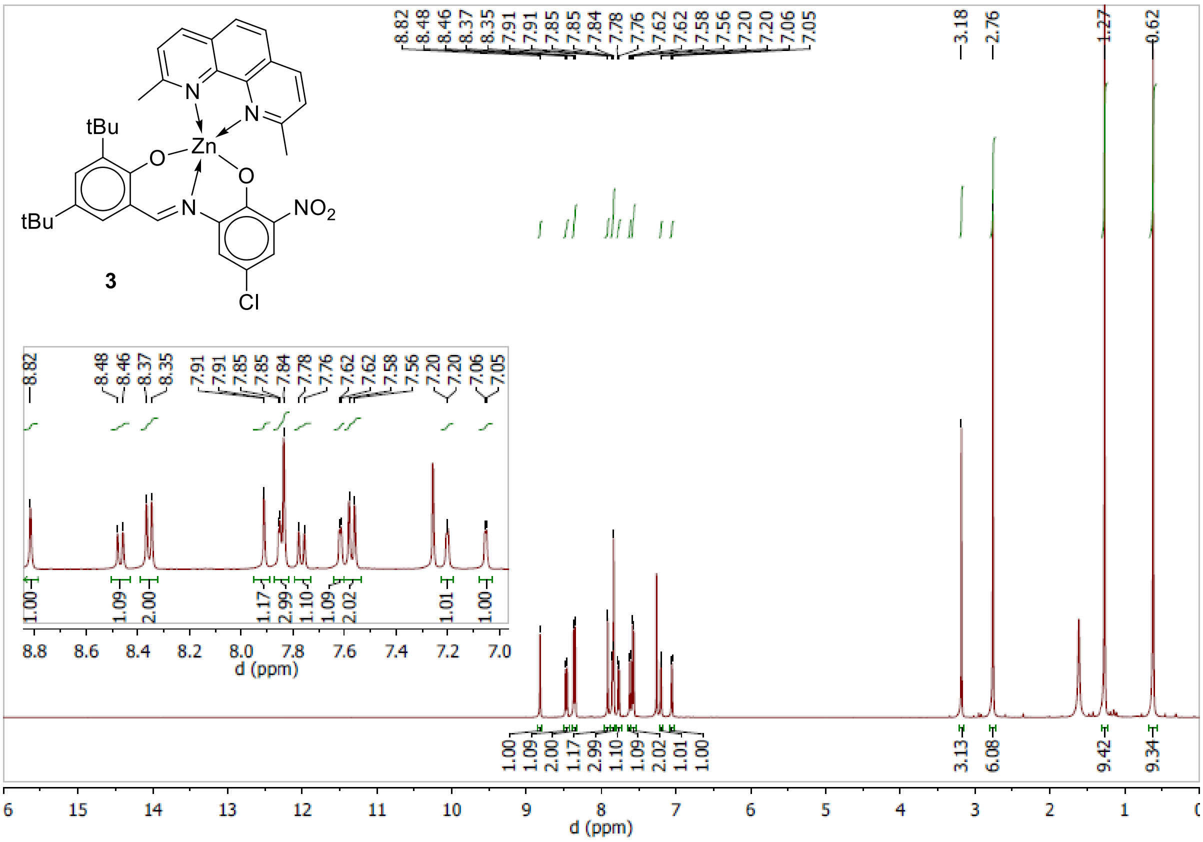

Figure S5. The ^{1}H NMR spectrum of **3**·0.5Neo (400 MHz, $CDCl_3$). The signals at 3.18, 7.77, 7.91, and 8.47 ppm belong to free ligand Neo (0.5 mol per 1.0 mol of **3**).

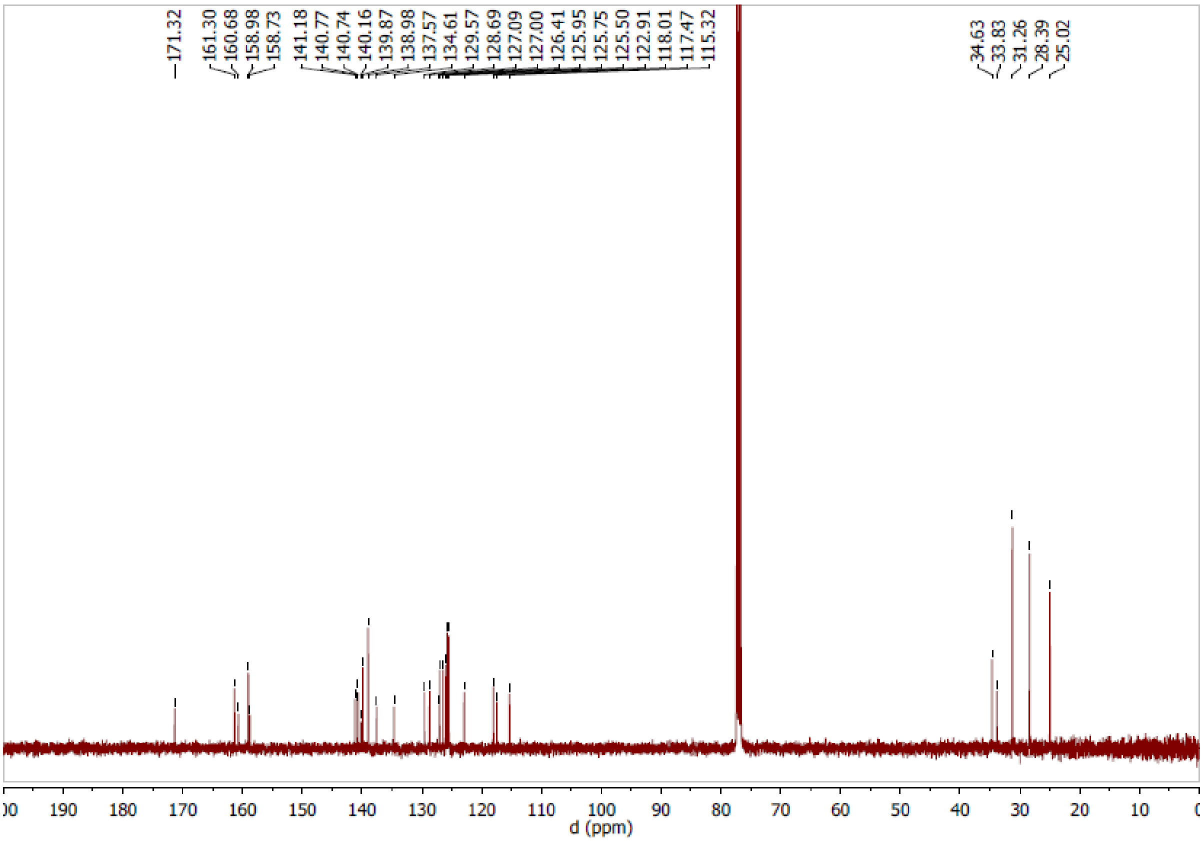

Figure S6. The $^{13}C\{^{1}H\}$ NMR spectrum of **3** (100 MHz, $CDCl_3$).

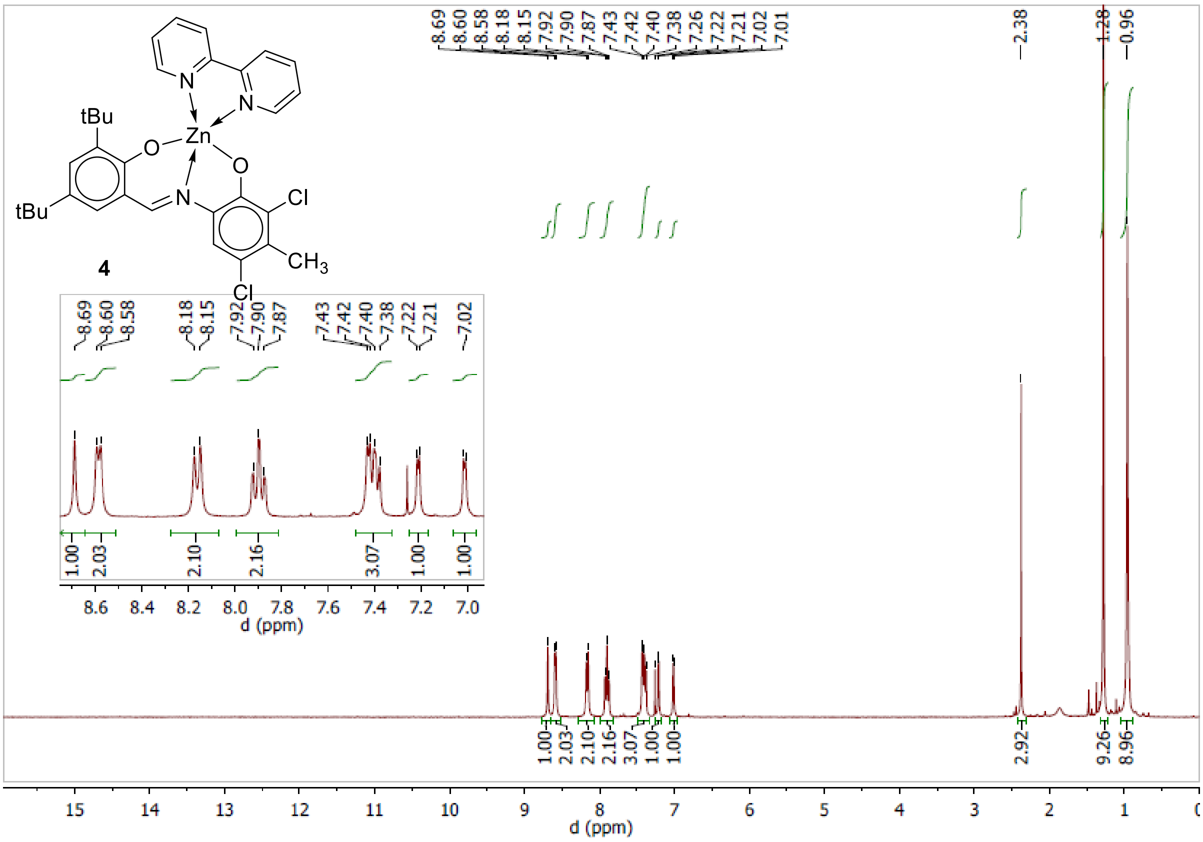

Figure S7. The ^{1}H NMR spectrum of **4** (400 MHz, $CDCl_3$).

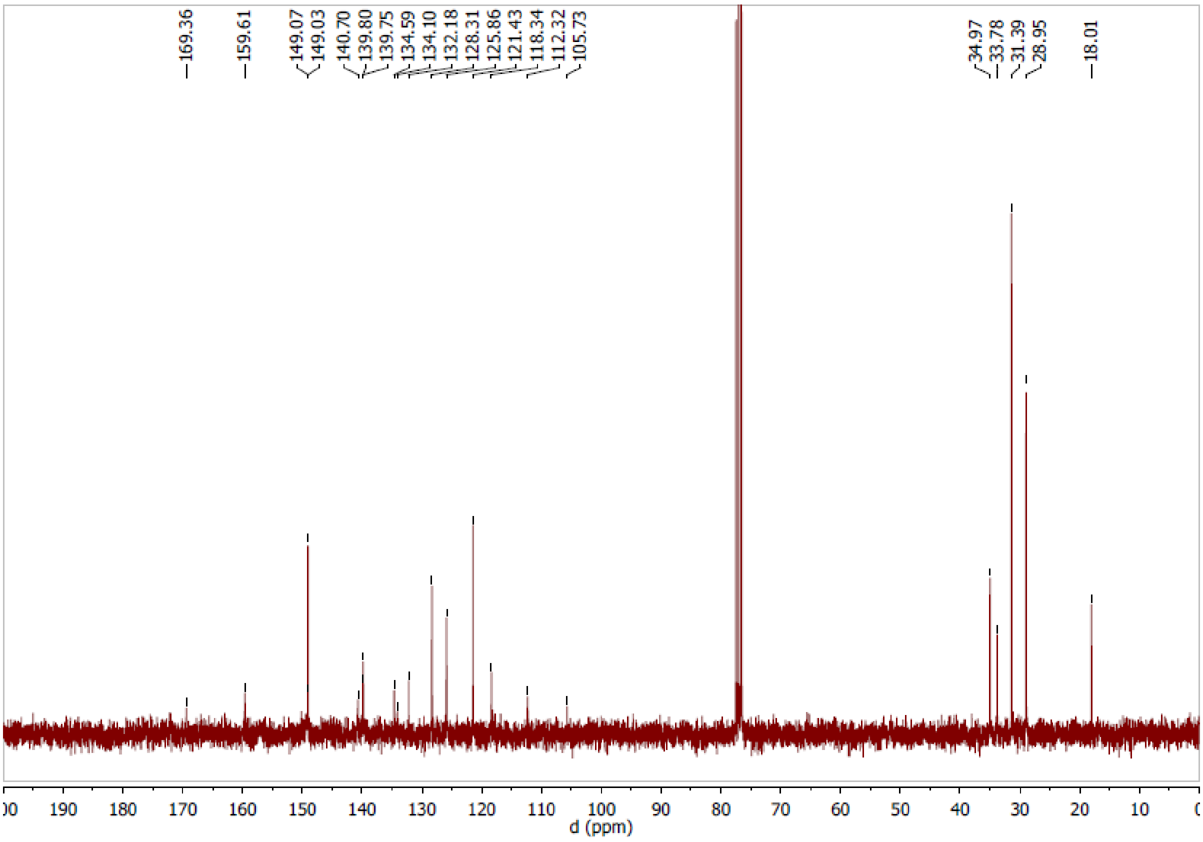

Figure S8. The $^{13}C\{^1H\}$ NMR spectrum of **4** (100 MHz, $CDCl_3$).

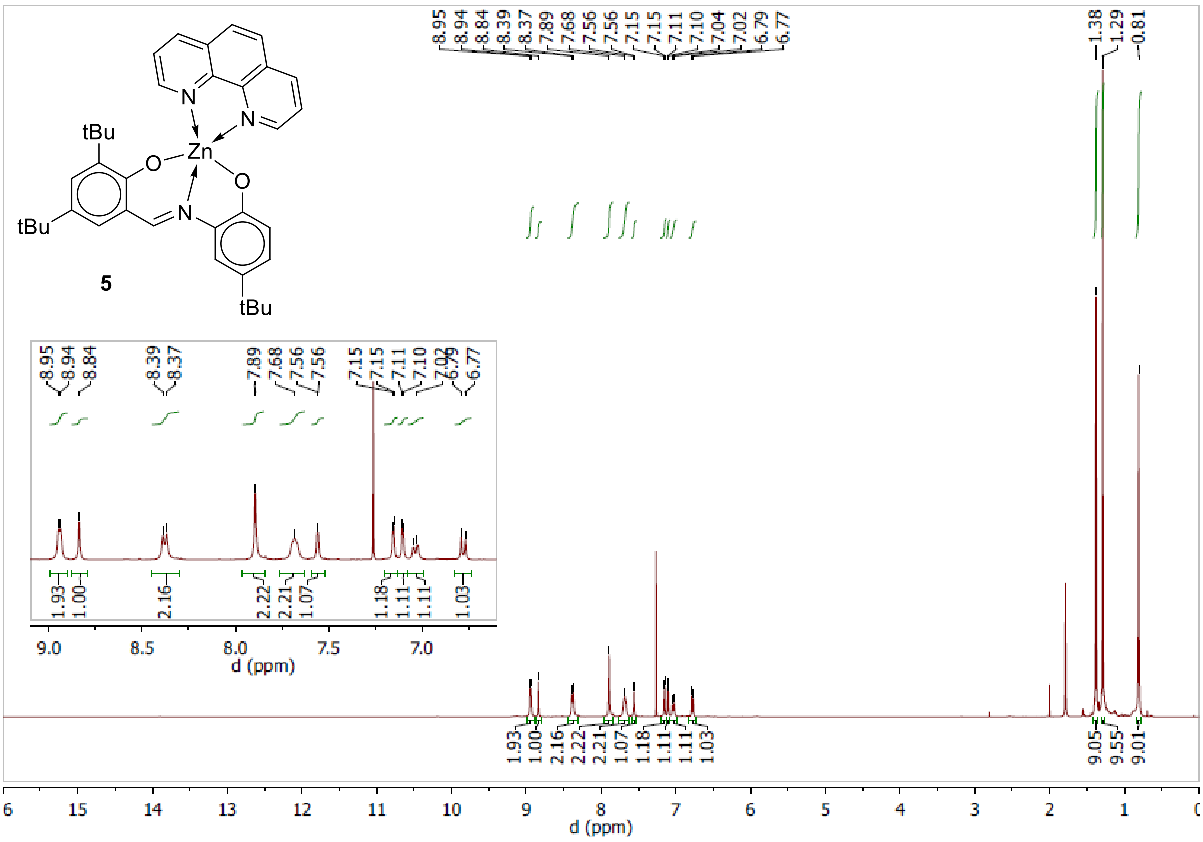

Figure S9. The ^{1}H NMR spectrum of **5** (400 MHz, $CDCl_3$).

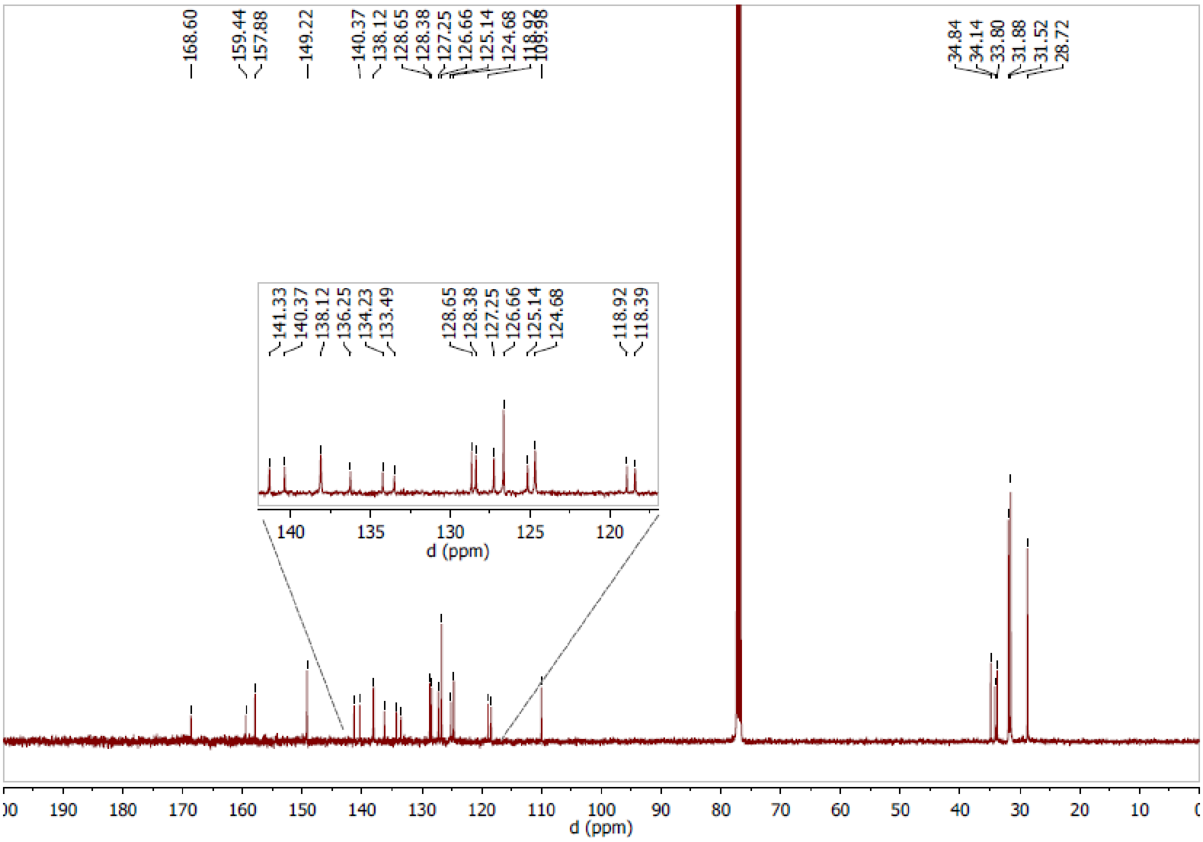

Figure S10. The $^{13}C\{^{1}H\}$ NMR spectrum of **5** (100 MHz, $CDCl_3$).

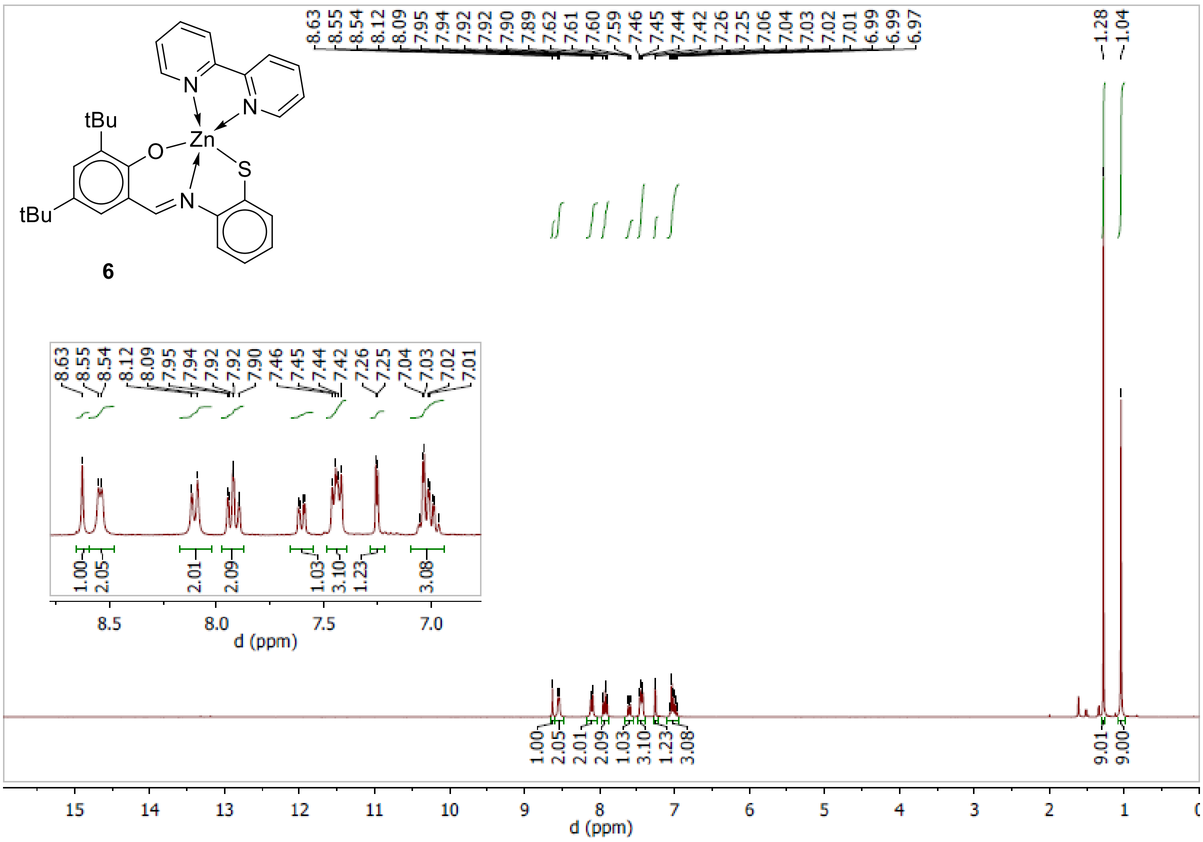

Figure S11. The ^{1}H NMR spectrum of **6** (400 MHz, $CDCl_3$).

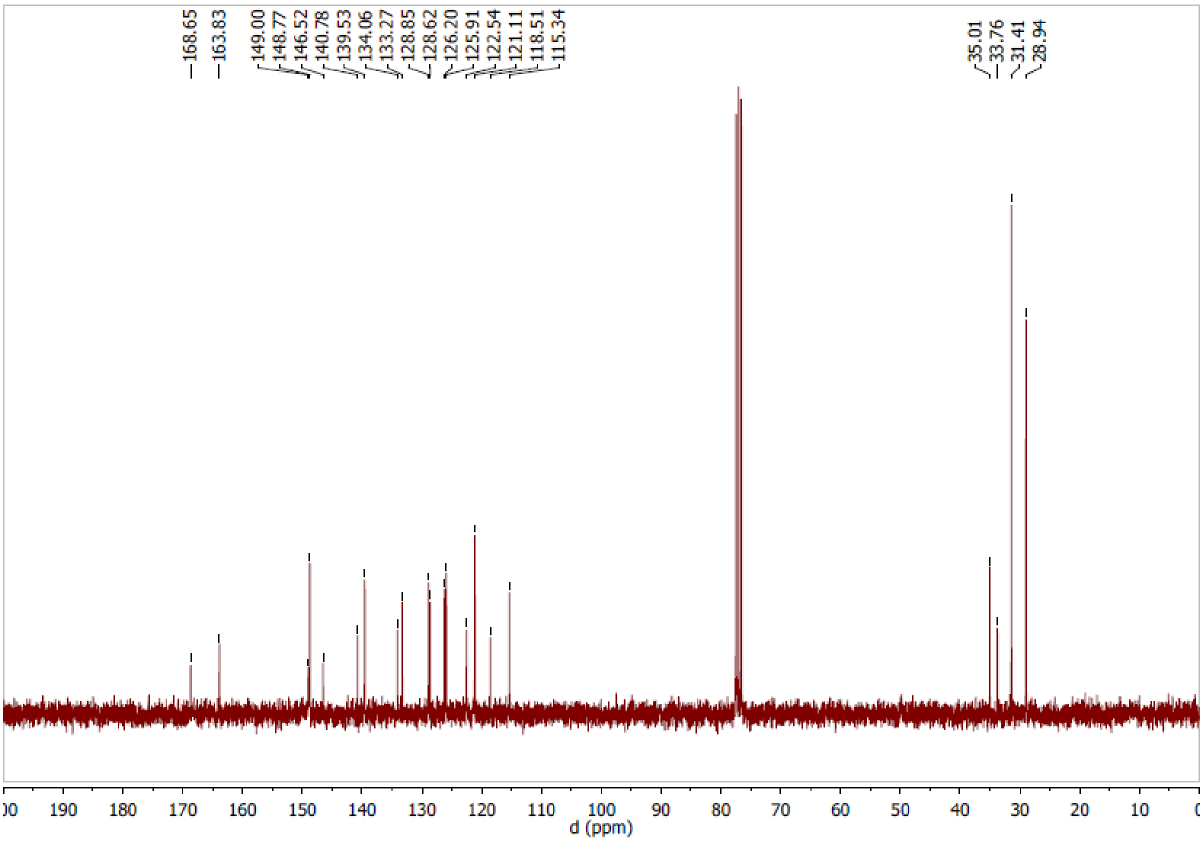

Figure S12. The $^{13}C\{^{1}H\}$ NMR spectrum of **6** (100 MHz, $CDCl_3$).

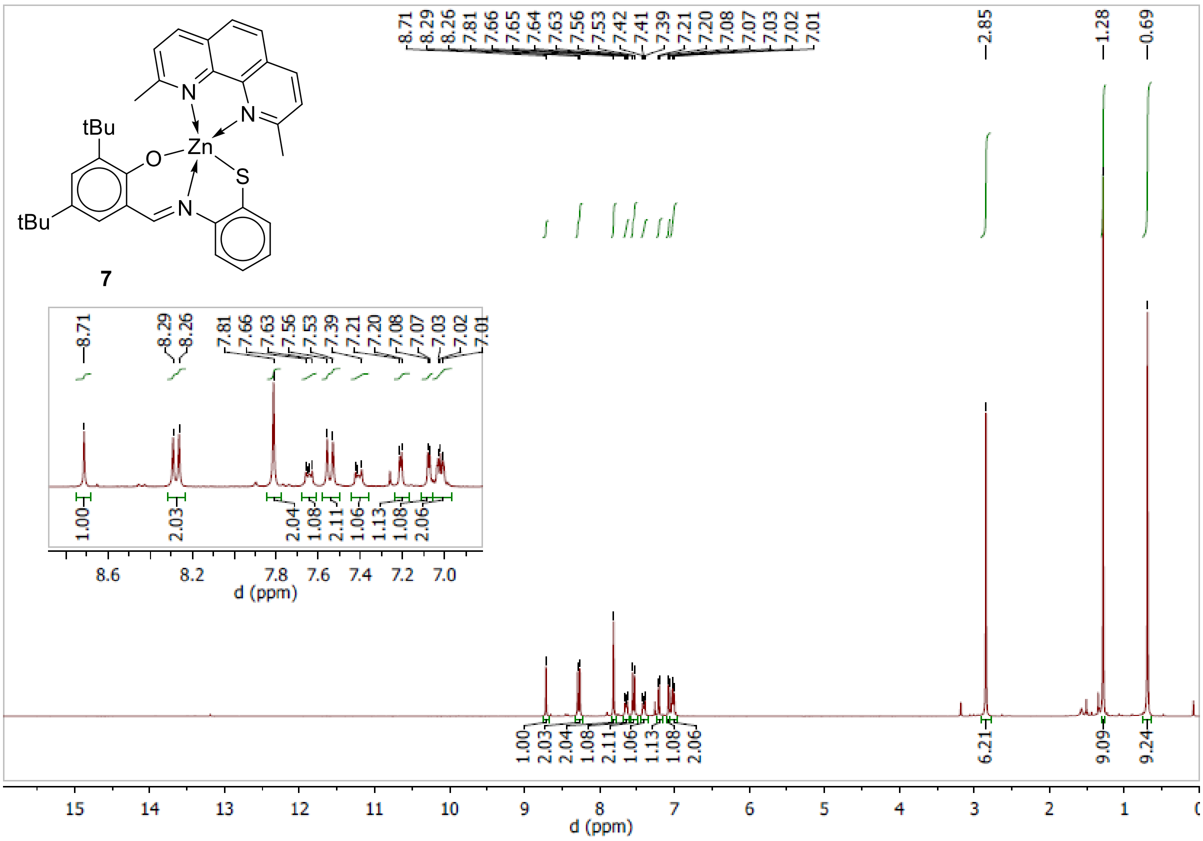

Figure S13. The ^{1}H NMR spectrum of **7** (400 MHz, $CDCl_3$).

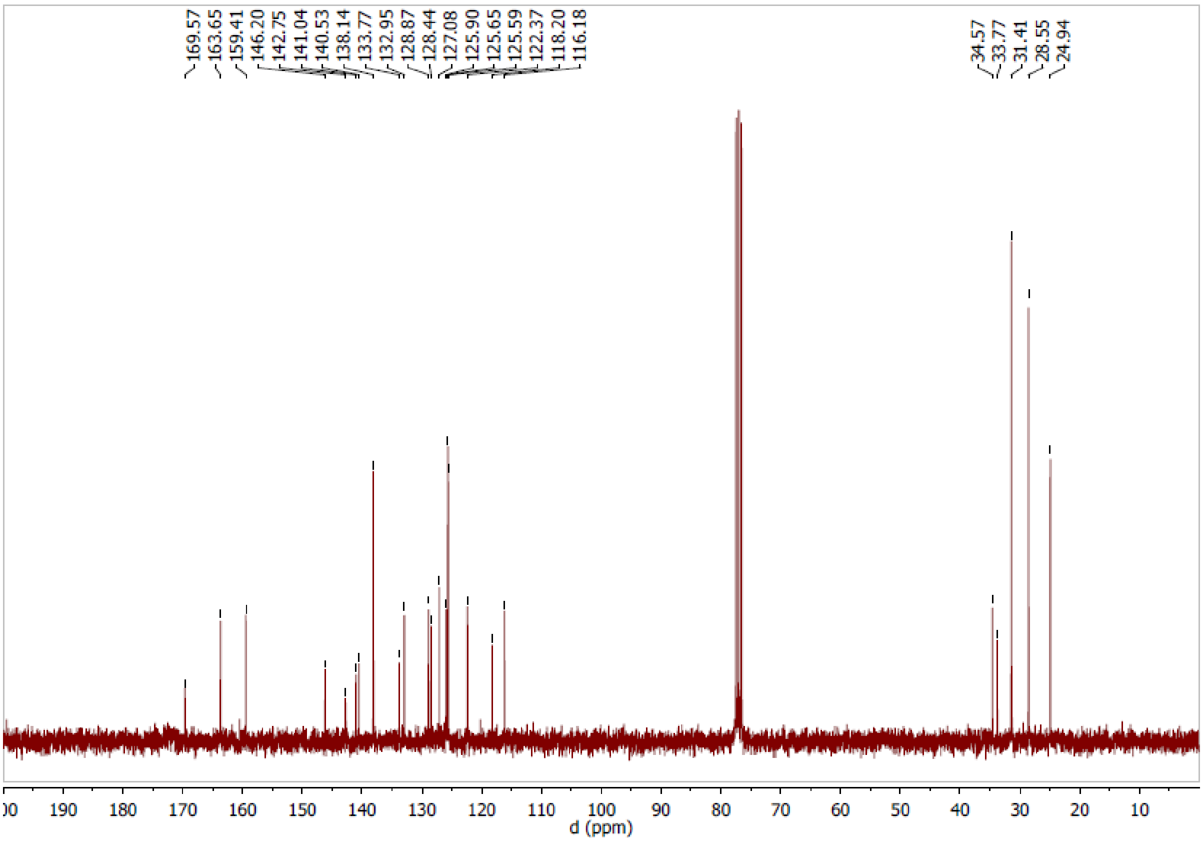

Figure S14. The ^{13}C{^{1}H} NMR spectrum of **7** (100 MHz, $CDCl_3$).

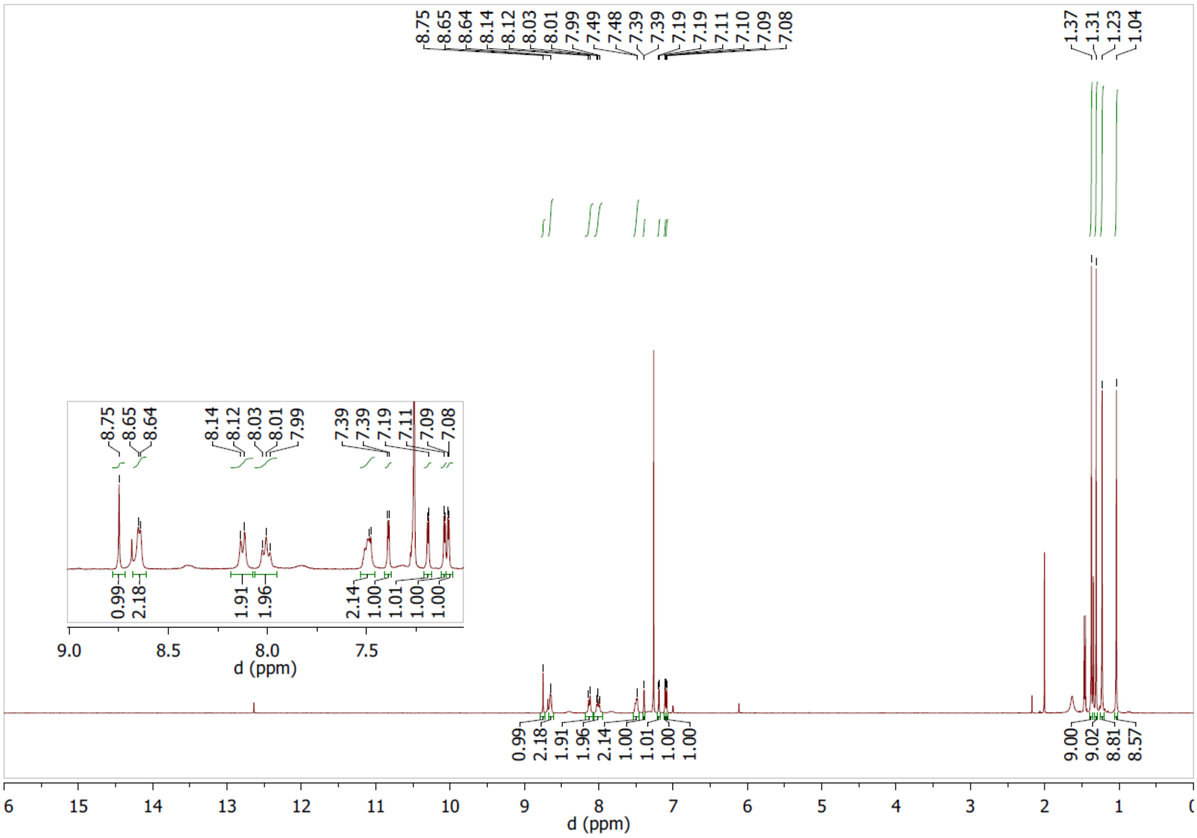

Figure S15. The ^{1}H NMR spectrum of **8** (400 MHz, $CDCl_3$).

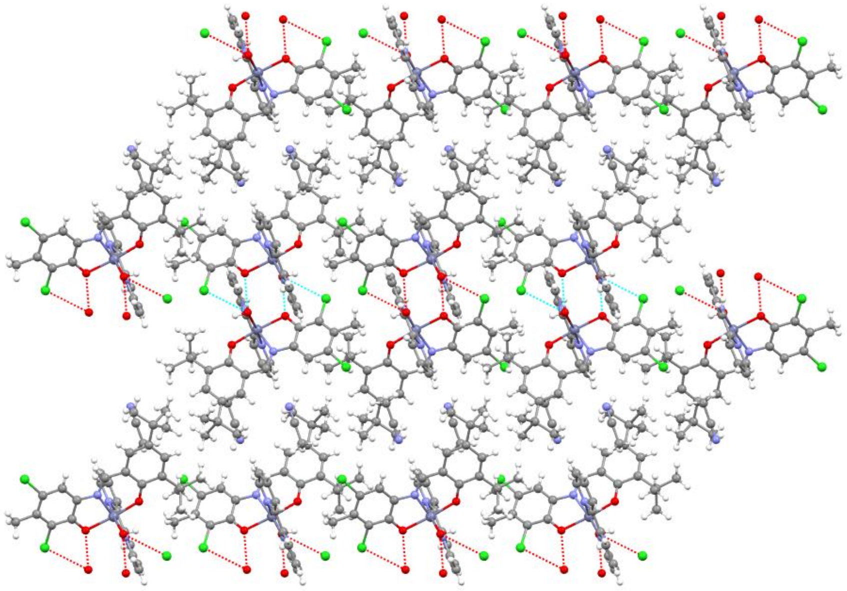

Figure S16. The crystal packing of **4**·H_2O·CH_3CN.

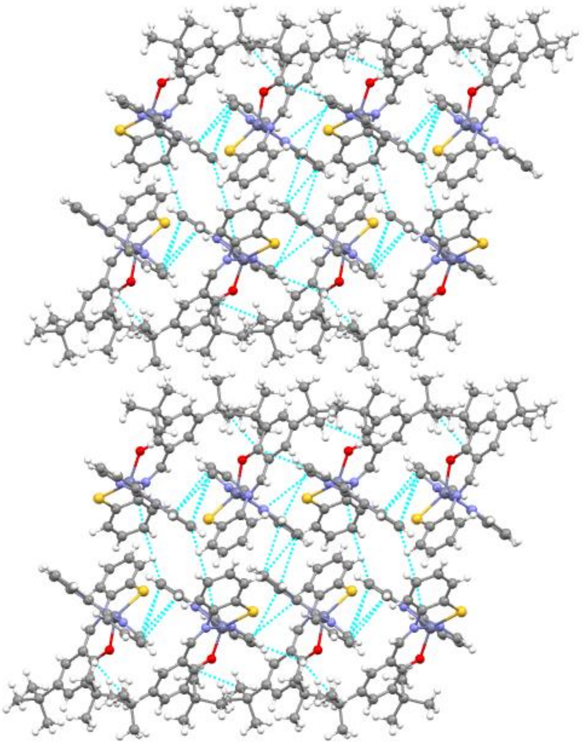

Figure S17. The crystal packing of **6**.

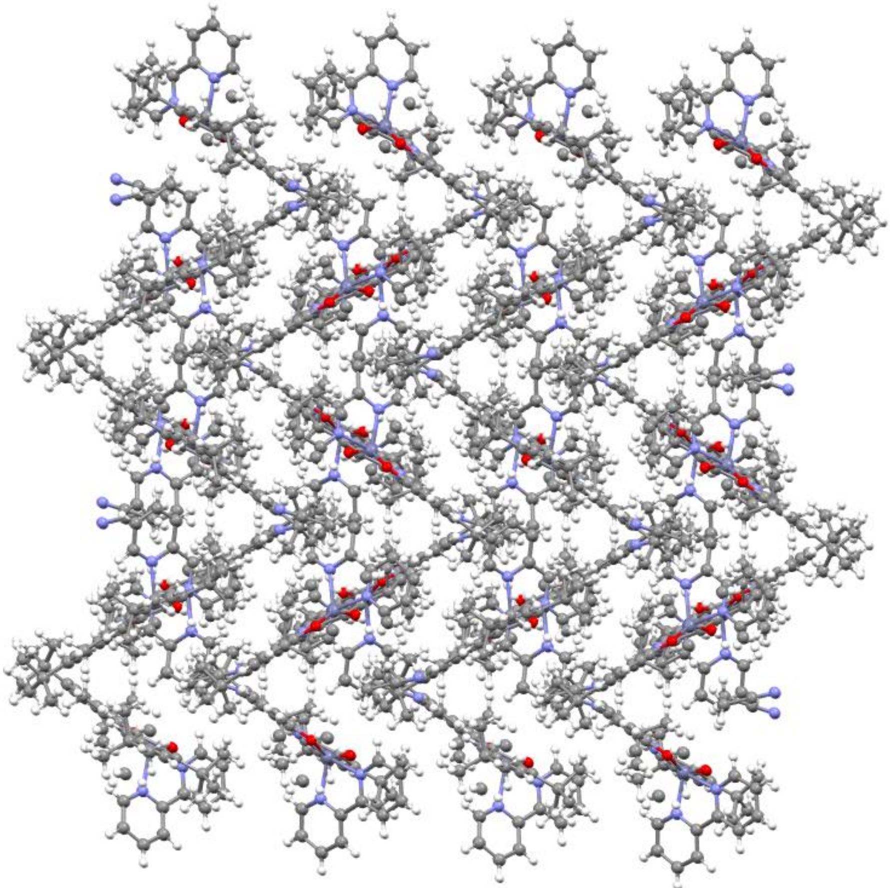

Figure S18. The crystal packing of **8**·CH_3CN.

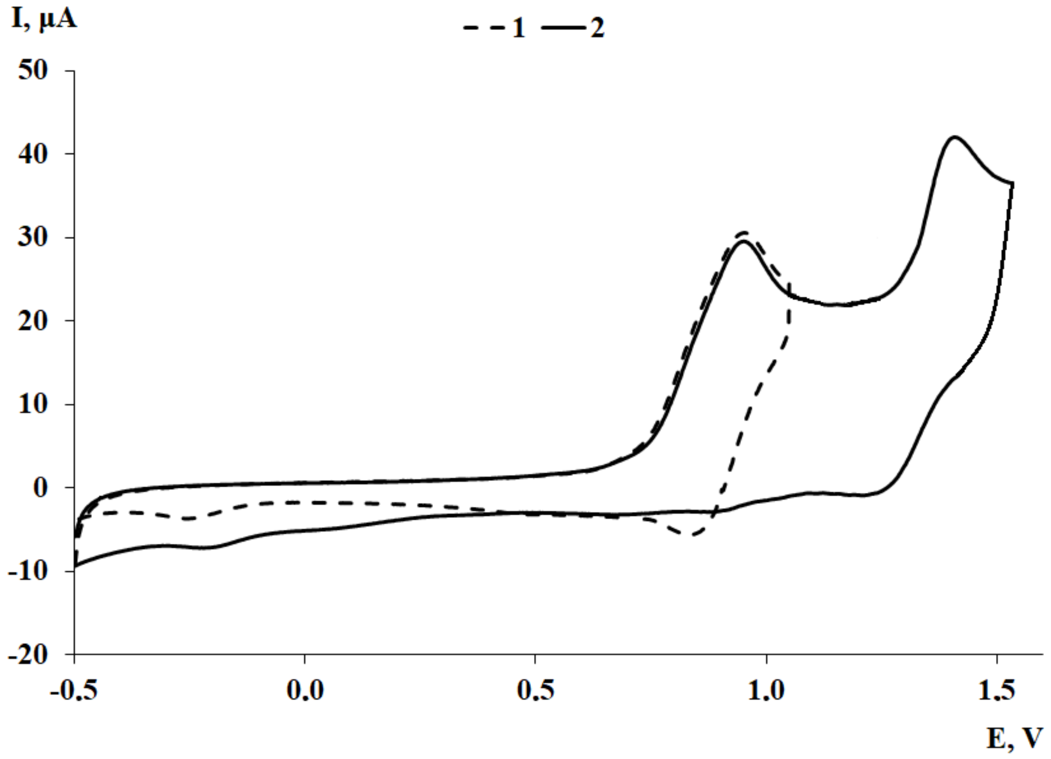

Figure S19. Cyclic voltammograms of **1** (L^1)Zn(Bipy) the potential switch from -0.50 to 1.0 V (curve 1); the potential switch from -0.50 to 1.55 V (curve 2) (CH_2Cl_2, C = 2 mmol, 0.1 M TBAP, scan rate 200 mV·s^{-1}).

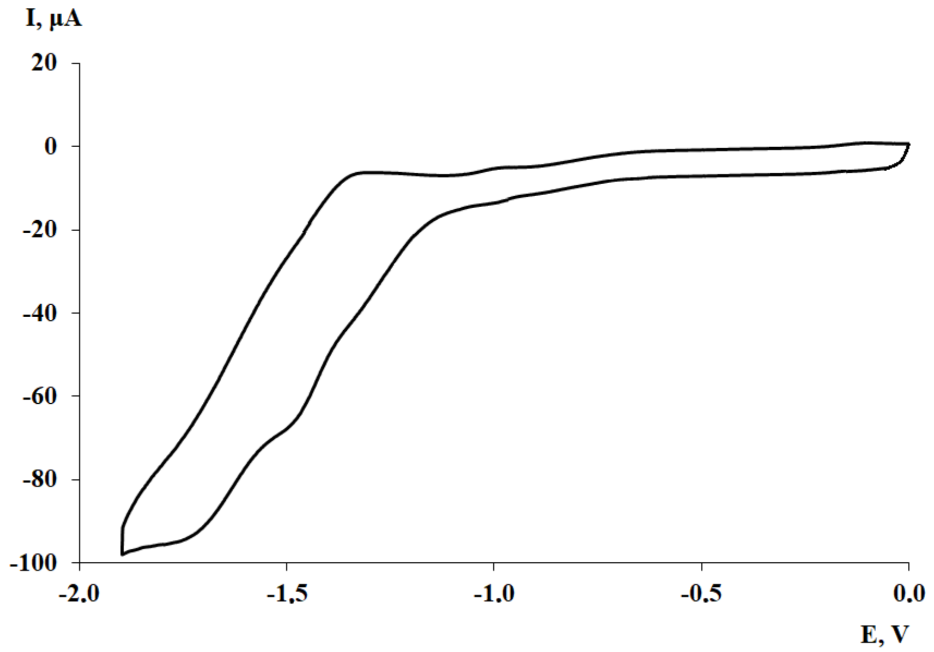

Figure S20. Cyclic voltammogram of **1** (L^1)Zn(Bipy) the potential switch from 0.0 to -1.9 V (CH_2Cl_2, C = 2 mmol, 0.1 M TBAP, scan rate 200 mV·s^{-1}).

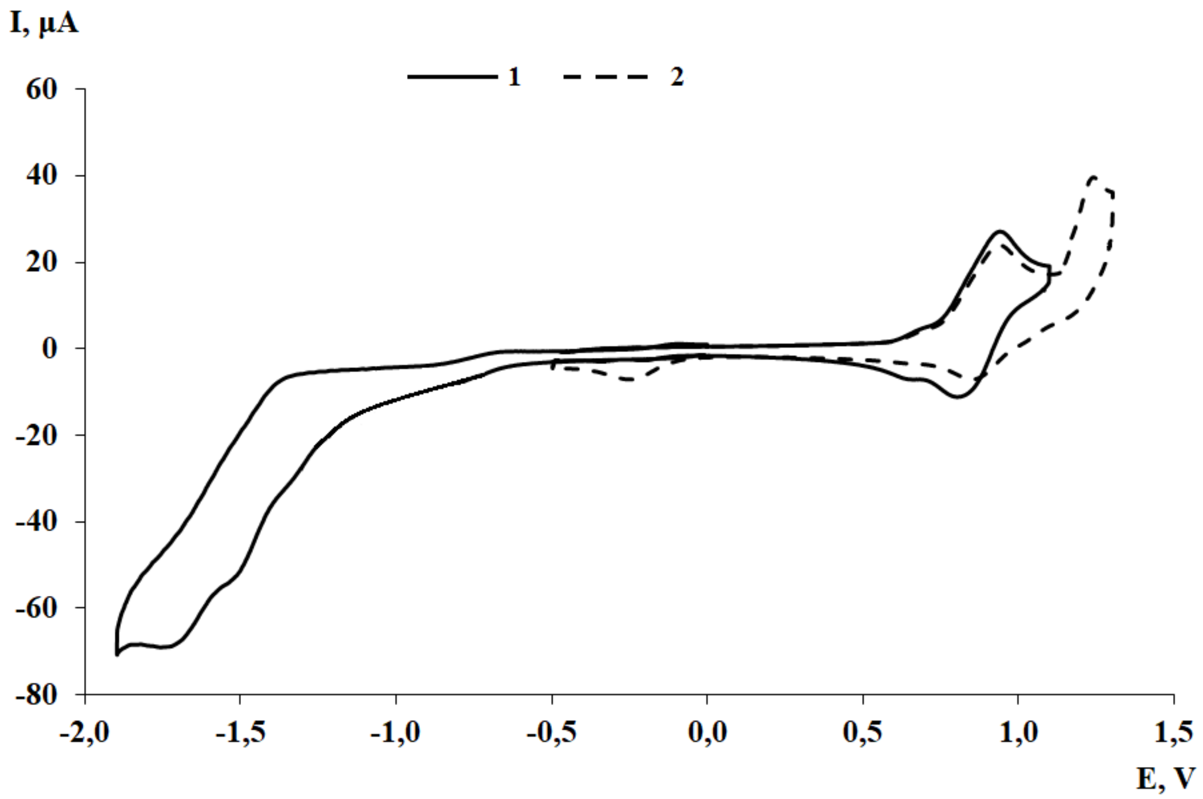

Figure S21. Cyclic voltammograms of **2** (L^1)Zn(Phen) the potential switch from 1.1 to -1.9 V (curve 1); the potential switch from -0.5 to 1.3 V (curve 2); (CH_2Cl_2, C = 2 mmol, 0.1 M TBAP, scan rate 200 mV·s^{-1}).

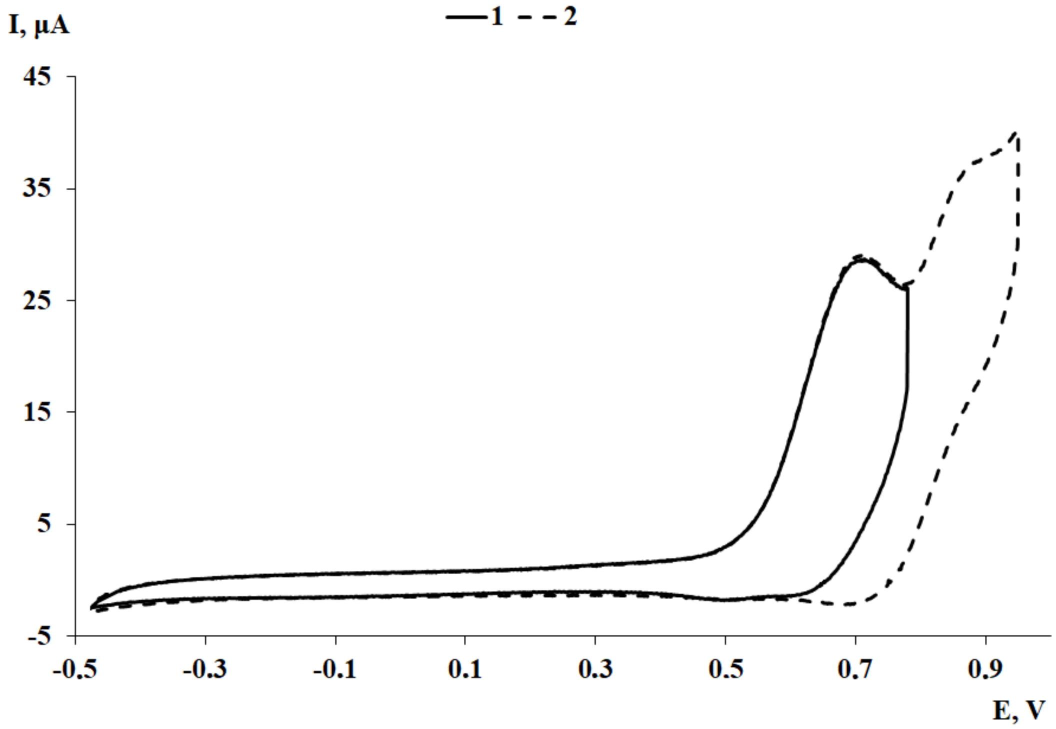

Figure S22. Cyclic voltammograms of **4** (L^2)Zn(Bipy) the potential switch from -0.45 to 0.80 V (curve 1); the potential switch from -0.45 to 0.95 V (curve 2); (CH_2Cl_2, C = 2 mmol, 0.1 M TBAP, scan rate 200 mV·s^{-1}).

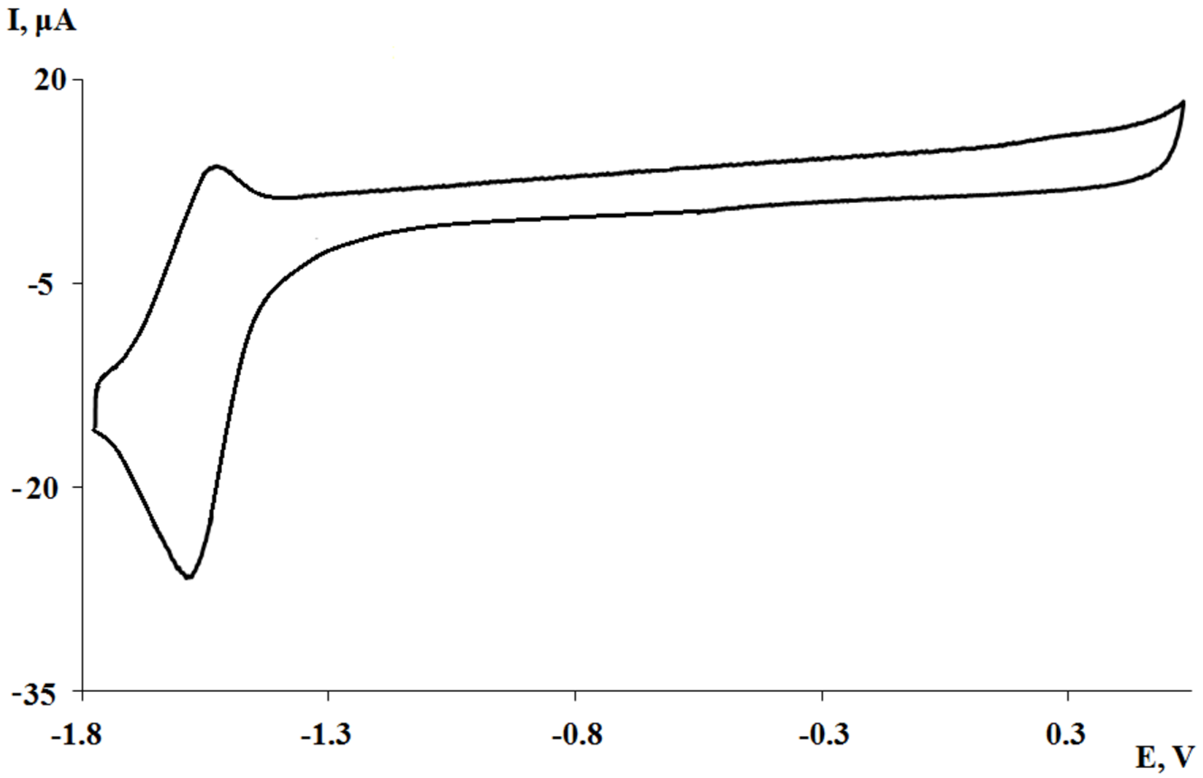

Figure S23. Cyclic voltammogram of **4** (L^2)Zn(Bipy) the potential switch from 0.50 to -1.75 V (CH_2Cl_2, C = 2 mmol, 0.1 M TBAP, scan rate 200 mV·s^{-1}).

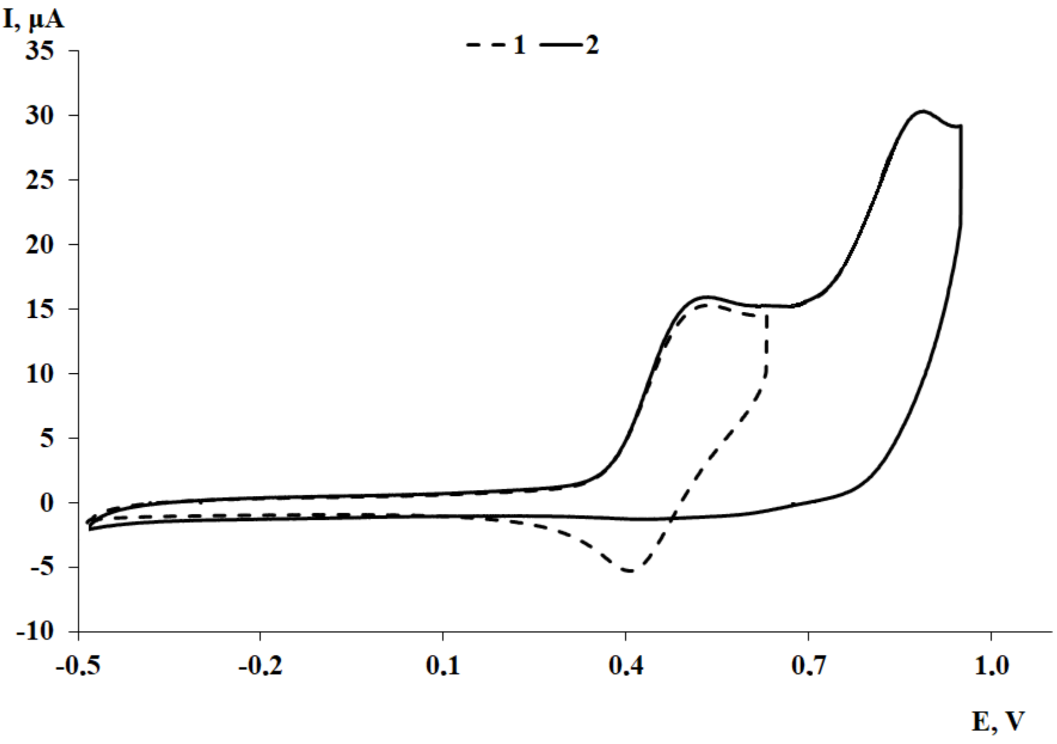

Figure S24. Cyclic voltammograms of **5** (L^3)Zn(Phen) the potential switch from -0.45 to 0.63 V (curve 1); the potential switch from -0.45 to 0.95 V (curve 2); (CH_2Cl_2, C = 2 mmol, 0.1 M TBAP, scan rate 200 mV·s^{-1}).

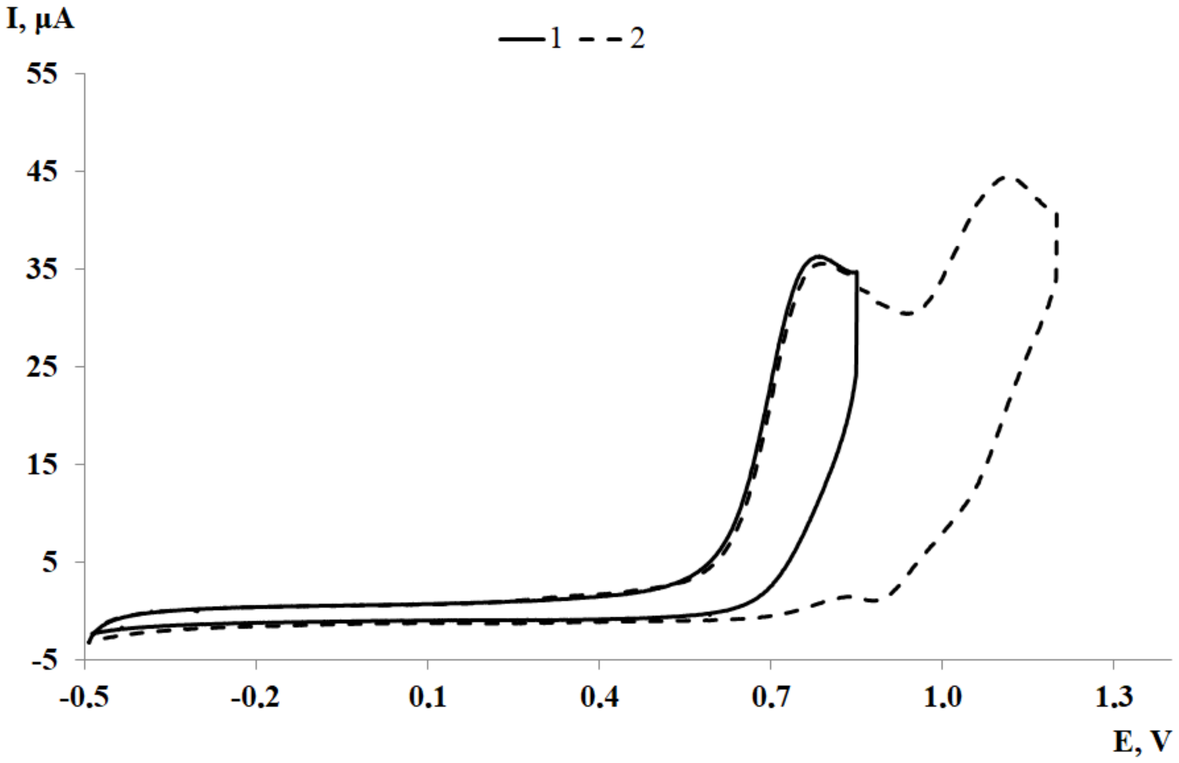

Figure S25. Cyclic voltammograms of **6** (L^5)Zn(Bipy) the potential switch from -0.50 to 0.85 V (curve 1); the potential switch from -0.50 to 1.20 V (curve 2); (CH_2Cl_2, C = 2 mmol, 0.1 M TBAP, scan rate 200 mV·s^{-1}).

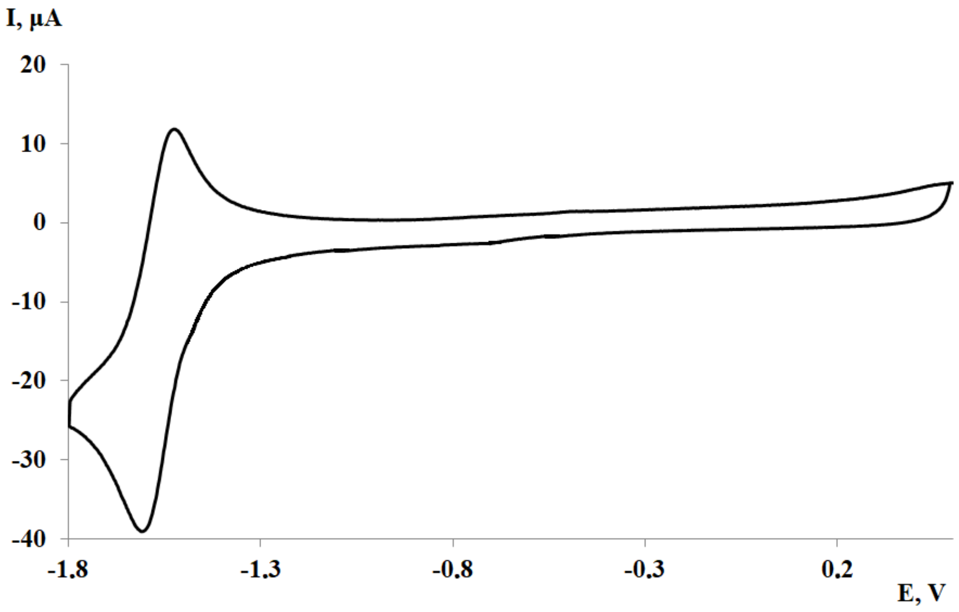

Figure S26. Cyclic voltammogram of **6** (L^5)Zn(Bipy) the potential switch from 0.50 to -1.80 V (CH_2Cl_2, C = 2 mmol, 0.1 M TBAP, scan rate 200 mV·s^{-1}).

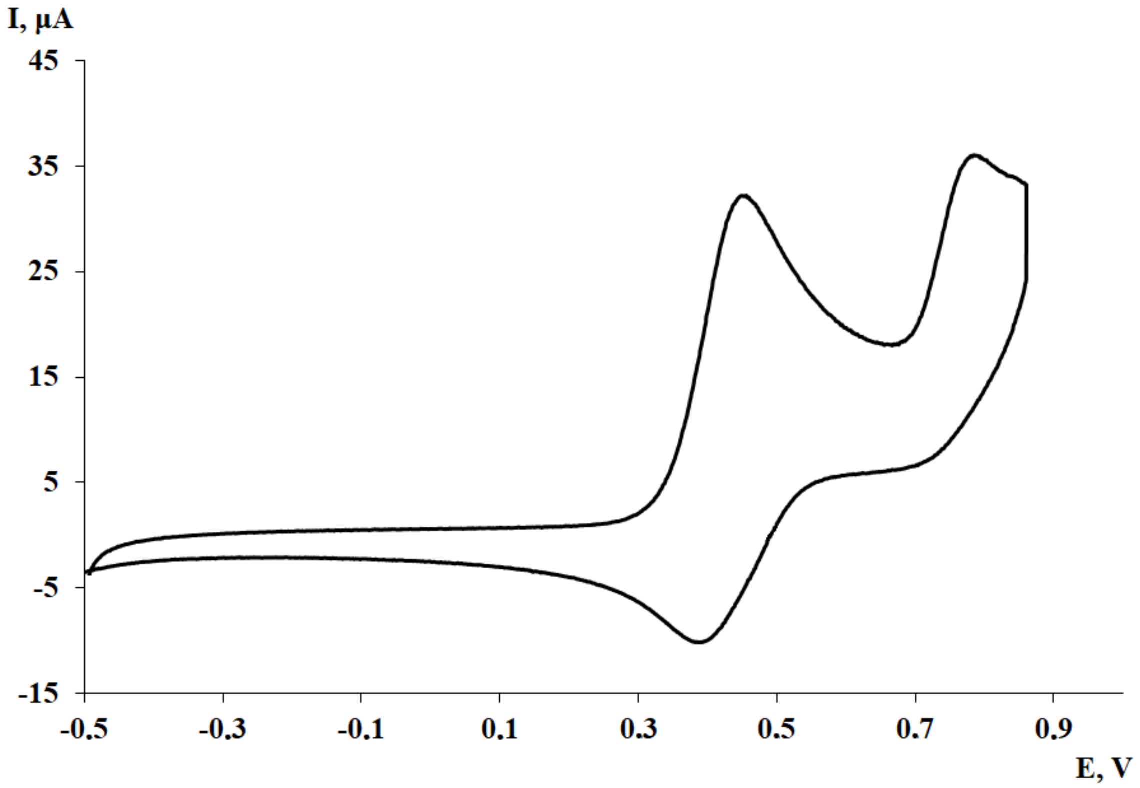

Figure S27. Cyclic voltammogram of **8** (L^4)Zn(Bipy) the potential switch from -0.50 to 0.85 V (CH_2Cl_2, C = 2 mmol, 0.1 M TBAP, scan rate 200 mV·s^{-1}).

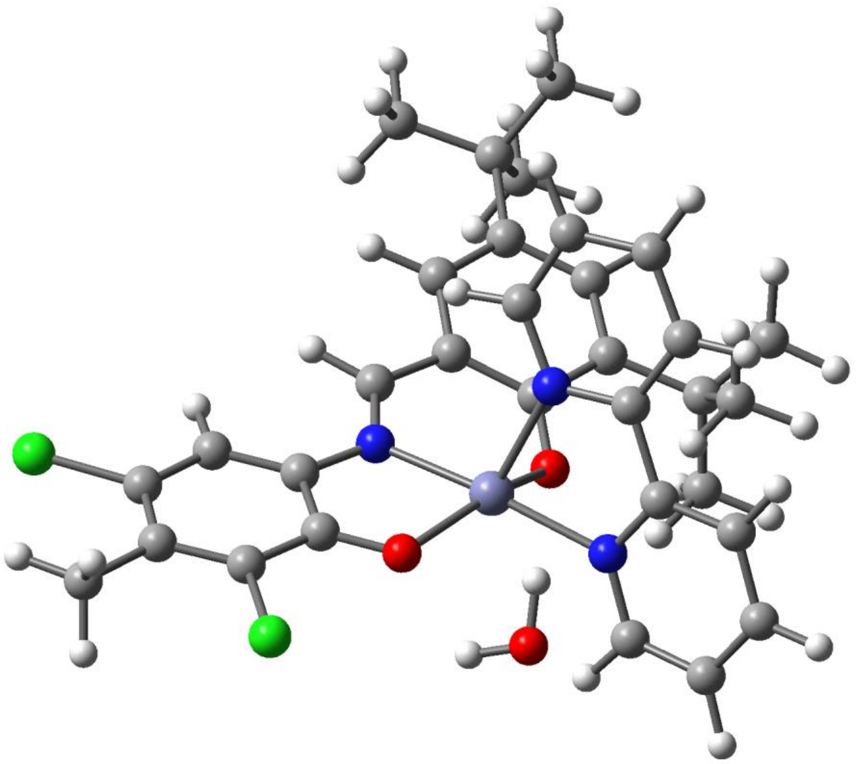

Figure S28. General view of the optimized molecular structure of **4**·H_2O.

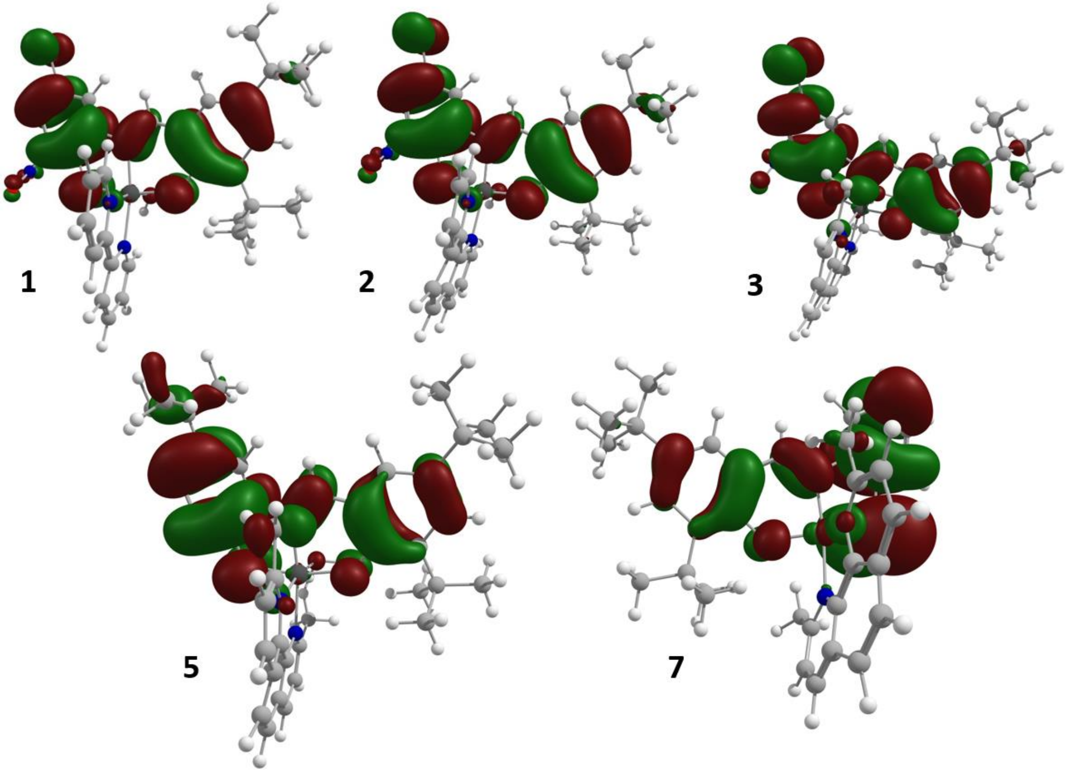

Figure S29. The isosurfaces of HOMO orbitals in the selected complexes.

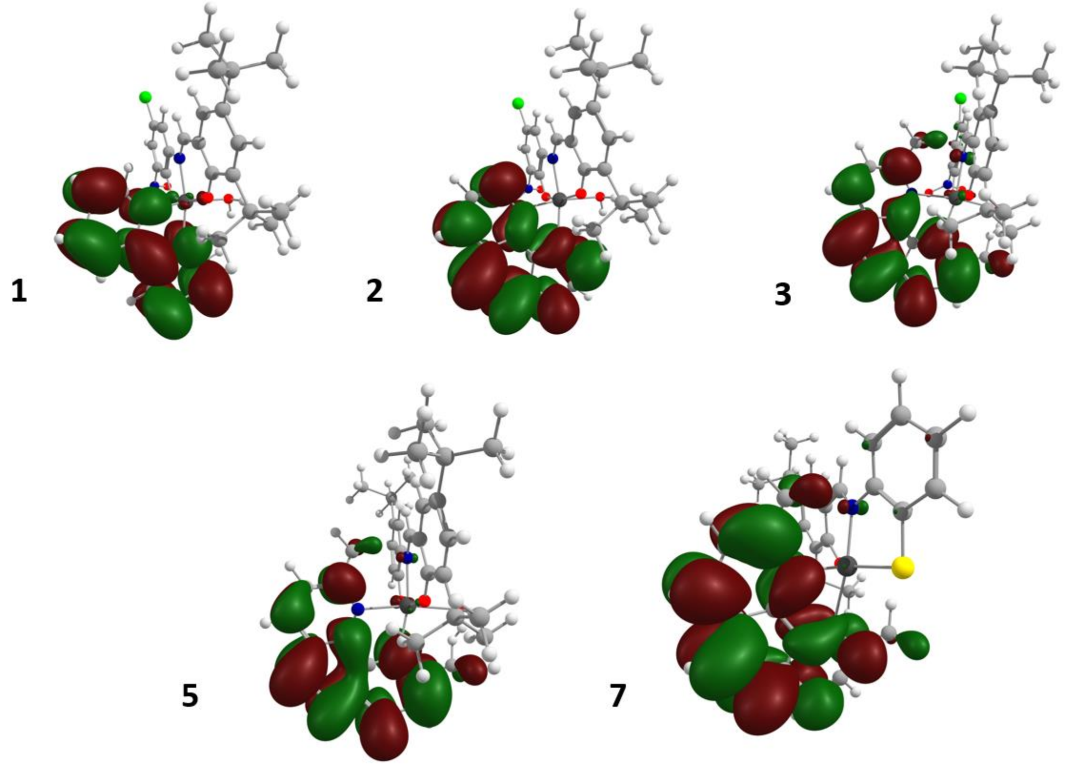

Figure S30. The isosurfaces of LUMO orbitals in the selected complexes.

Table S1. H-bonds in crystals of investigated compounds.

H-bond	Symmetry generation	D-H, Å	H...A, Å	D...A, Å	D-H...A, °
**4**					
O3-H3A...O1	2-x,1-y,1-z	0.87	1.94	2.681(3)	141
O3-H3B...Cl61	2-x,1-y,1-z	0.87	2.69	3.302(2)	128
C51-H51C...Cl41	x,y,z	0.98	2.56	3.086(4)	114
C92-H92A...O2	x,y,z	0.98	2.27	2.936(4)	124
C93-H93C...O2	x,y,z	0.98	2.39	3.040(4)	123
**6**					
C3A-H3A...S1B	1+x,y,z	0.95	2.87	3.810(4)	173
C17B-H17B...S1A	x,-1+y,z	0.95	2.81	3.491(4)	129
C92B-H92F...O2B	x,y,z	0.98	2.36	3.009(5)	123
C93A-H93C...O2A	x,y,z	0.98	2.27	2.943(5)	125
C93B-H93D...O2B	x,y,z	0.98	2.28	2.943(5)	124
C94A-H94A...O2A	x,y,z	0.98	2.38	3.034(4)	123
**8**					
C21-H21...O2	1/2-x,1/2+y,z	0.95	2.35	3.293(3)	169
C63-H63A...O1	x,y,z	0.98	2.34	3.007(4)	125
C64-H64C...O1	x,y,z	0.98	2.34	3.023(4)	126
C93-H93A...O2	x,y,z	0.98	2.35	2.989(4)	122
C94-H94C...O2	x,y,z	0.98	2.38	3.033(4)	124

Table S2. The details of X-ray experiment and structure refinement

Complex	**4**·H_2O·CH_3CN	**6**	**8**·CH_3CN
Empirical formula	$C_{34}H_{38}Cl_2N_4O_3Zn$	$C_{31}H_{33}N_3OSZn$	$C_{41}H_{52}N_4O_2Zn$
Formula weight	686.95	561.03	698.23
T / K	150(2)	150(2)	100(2)
Crystal system	monoclinic	triclinic	orthorhombic
Space group	P21/c	P -1	Pbca
a / Å	17.0046(8)	11.2220(8)	17.7089(6)
b / Å	9.3695(6)	12.1251(9)	16.1000(6)
c / Å	20.9772(10)	21.4863(16)	27.2185(8)
α / °	90	93.243(3)	90
β / °	90.147(2)	93.314(3)	90
γ / °	90	91.458(3)	90
V / $Å^3$	3342.2(3)	2912.8(4)	7760.4(5)
Z	4	4	8
ρ / g·cm^{-3}	1.365	1.279	1.195
μ / mm^{-1}	0.934	0.942	0.671
F(000)	1432	1176	2976
Q range / °	1.942 - 26.000	1.818 - 25.999	1.866 - 25.999
Reffl. Collected	6570	11421	7619
Reffl. unique	4621	9062	6459
R_{int}	0.1093	0.0321	0.0606
GOOF (F^2)	0.956	1.065	1.096
R_1 / wR_2 ($I > 2\sigma(I)$)	0.0468 / 0.0956	0.0516 / 0.1246	0.0504 / 0.1228
R_1 / wR_2 (all data)	0.0747 / 0.1058	0.0695 / 0.1339	0.0603 / 0.1279
Largest diff. peak and hole / e·$Å^3$	0.459 and -0.536	1.254 and -0.455	0.896 and -0.471
